# Reducing Inequities During the COVID-19 Pandemic: A Rapid Review and Synthesis of Public Health Recommendations

**DOI:** 10.3389/phrs.2021.1604031

**Published:** 2022-01-17

**Authors:** Chloe Brown, Katie Wilkins, Amy Craig-Neil, Tara Upshaw, Andrew David Pinto

**Affiliations:** ^1^ Upstream Lab, MAP/Centre for Urban Health Solutions, Li Ka Shing Knowledge Institute, Unity Health Toronto, Toronto, ON, Canada; ^2^ Undergraduate Medical Education, Faculty of Medicine, University of Toronto, Toronto, ON, Canada; ^3^ Institute of Health Policy Management and Evaluation, University of Toronto, Toronto, ON, Canada; ^4^ Dalla Lana School of Public Health, University of Toronto, Toronto, ON, Canada; ^5^ Department of Family and Community Medicine, Faculty of Medicine, University of Toronto, Toronto, ON, Canada; ^6^ Department of Family and Community Medicine, St. Michael’s Hospital, Toronto, ON, Canada

**Keywords:** public health, social determinants of health, SARS-CoV-2, health equity, COVID-19, prevention

## Abstract

**Objectives:** Efforts to contain the COVID-19 pandemic should take into account worsening health inequities. While many public health experts have commented on inequities, no analysis has yet synthesized recommendations into a guideline for practitioners. The objective of this rapid review was to identify the areas of greatest concern and synthesize recommendations.

**Methods:** We conducted a rapid systematic review (PROSPERO: CRD42020178131). We searched Ovid MEDLINE, Embase, PsycINFO, CINAHL and Cochrane Central Register of Controlled Trials databases from December 1, 2019 to April 27, 2020. We included English language peer-reviewed commentaries, editorials, and opinion pieces that addressed the social determinants of health in the context of COVID-19.

**Results:** 338 articles met our criteria. Authors represented 81 countries. Income, housing, mental health, age and occupation were the most discussed social determinants of health. We categorized recommendations into primordial, primary, secondary and tertiary prevention that spoke to the social determinants of COVID-19 and equity.

**Conclusion:** These recommendations can assist efforts to contain COVID-19 and reduce health inequities during the pandemic. Using these recommendations, public health practitioners could support a more equitable pandemic response.

**Systematic Review Registration**: PROSPERO, CRD42020178131.

## Introduction

SARS-CoV-2 emerged as a novel pathogen in late 2019 and quickly became a leading cause of morbidity and mortality worldwide [1]. In over 1 year, the COVID-19 pandemic spread globally and resulted in over 150 million confirmed cases and over 3 million deaths [2]. Efforts to contain the virus have caused economic recessions [3], halted global travel and trade, and required the mass closure of places of employment and education [4]. Initially called a “great equalizer” [5], experts in public health quickly recognized that the COVID-19 pandemic and its aftermath disproportionately impacts individuals and communities that had previously been made vulnerable by the social determinants of health (SDoH).

Reports emerged in March 2020 that COVID-19 outcomes were associated with key SDoH. Data from Canada, the United States, and the United Kingdom indicated higher rates of COVID-19 infections, hospital admissions, and mortality in low-income areas with high household density [6]. Racialized communities in these countries were particularly impacted [7], likely due to systemic racism that affects occupation and work, socioeconomic status, access to health care, and housing. The risk of COVID-19 varied by neighbourhood [7], housing status (e.g., homeless shelters, prisons) [8, 9], type of work (e.g., essential services, retail) [10], and income [7].

Numerous experts have provided recommendations on how public health officials and policymakers should consider the SDoH of COVID-19. The objective of this rapid review is to identify the areas of greatest concern and synthesize recommendations to support a more equitable pandemic response.

## Methods

We conducted a rapid review following Cochrane Rapid Review Methods Group [11] and PRISMA guidelines [12]. This review is registered with PROSPERO (CRD42020178131).

### Search Strategy and Selection Criteria

We searched Ovid MEDLINE, Embase, PsycINFO, CINAHL and Cochrane Central Register of Controlled Trials bibliographic databases from December 1, 2019 to April 1, 2020, updating the search on April 16, 2020 and on April 27, 2020 (Supplementary Appendix S1). We included English language commentaries, editorials, analyses and opinion pieces from peer-reviewed journals that discussed COVID-19 in relation to equity, the social determinants of health, and/or vulnerable populations. Using the WHO Commission on the Social Determinants of Health framework [13] we included articles that discussed the following SDoH: age, disability, education, food security, gender, governance, housing, immigration status, income, mental health, occupation, race and ethnicity, rural/urban geography, sexual orientation, and social isolation or social capital. We excluded quantitative original research (separately analysed in PLOS ONE [14]), qualitative original research, reviews, and mixed-methods studies.

### Publication Selection

Titles, abstracts, and full-text (where necessary) were screened against our inclusion and exclusion criteria using DistillerSR citation management software (Evidence Partners, Ottawa, Canada). Following rapid systematic review methods, 80% of retrieved records were single screened by one team of three independent reviewers, and 20% were double screened by a second team of five independent reviewers. The second team verified exclusion decisions for single-reviewed records. A third team resolved conflicts for double-reviewed records by deliberation. Following this initial review, two authors conducted a secondary full text review of all included articles.

### Data Extraction, Quality Appraisal, and Synthesis

After finalizing the included articles, the study team independently reviewed 20 randomly selected articles to create a data extraction table and initial coding framework. Through meetings, this coding framework was refined to support our narrative analysis and synthesis. We organized recommendations by SDoH, and into primordial, primary, secondary, and tertiary prevention [15]. Primordial prevention focuses on factors that reduce foundational risk factors (e.g., psychosocial, environmental), primary prevention prevents onset of disease by altering behaviours and exposures, secondary prevention focuses on detecting and treating early stages of disease, and tertiary prevention focuses on mitigating the impact of disease on those infected [15–17]. We also analyzed the country of origin of authors, article type, and type of evidence used. Three authors individually completed data extraction, coding, and quality assessment (Supplementary Appendix S2). We used the Joanna Briggs Institute Critical Appraisal Tool for text and opinion studies for quality appraisal [18]. Due to the heterogenous nature of the articles, a metanalysis could not be completed, so the studies were synthesized using a narrative approach.

## Results

**FIGURE 1:**
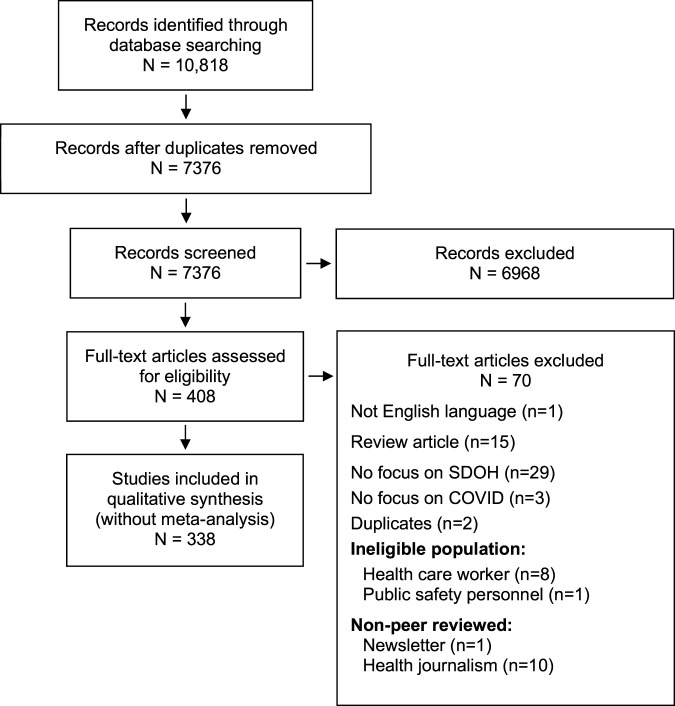
PRISMA flow diagram (Systematic Review, Global, 2019-2020).

A total of 7,376 citations were screened (Figure 1), of which 338 articles met our inclusion criteria (Supplementary Appendix S3). The authors represent 81 countries from Africa, Asia, Australia, Europe, North America, and South America. The majority of the articles analyzed were of good quality using the Joanna Briggs Institute Critical Appraisal Tool. In 327 (96.7%) of the articles, the source of the opinions was easily identifiable. In 292 (86.4%) articles, the identified source of the opinion had standing in the relevant field of expertise. In 313 (92.6%) articles, the best interests of the population being discussed was the central focus. In 314 (92.9%) articles, the opinions discussed were presented logically, and seemed to be the result of analytical thought. In 269 (79.6%) articles, relevant literature in the field was referred to. In 182 (53.8%) articles, inconsistencies with the literature were identified and logically defended.

**FIGURE 2:**
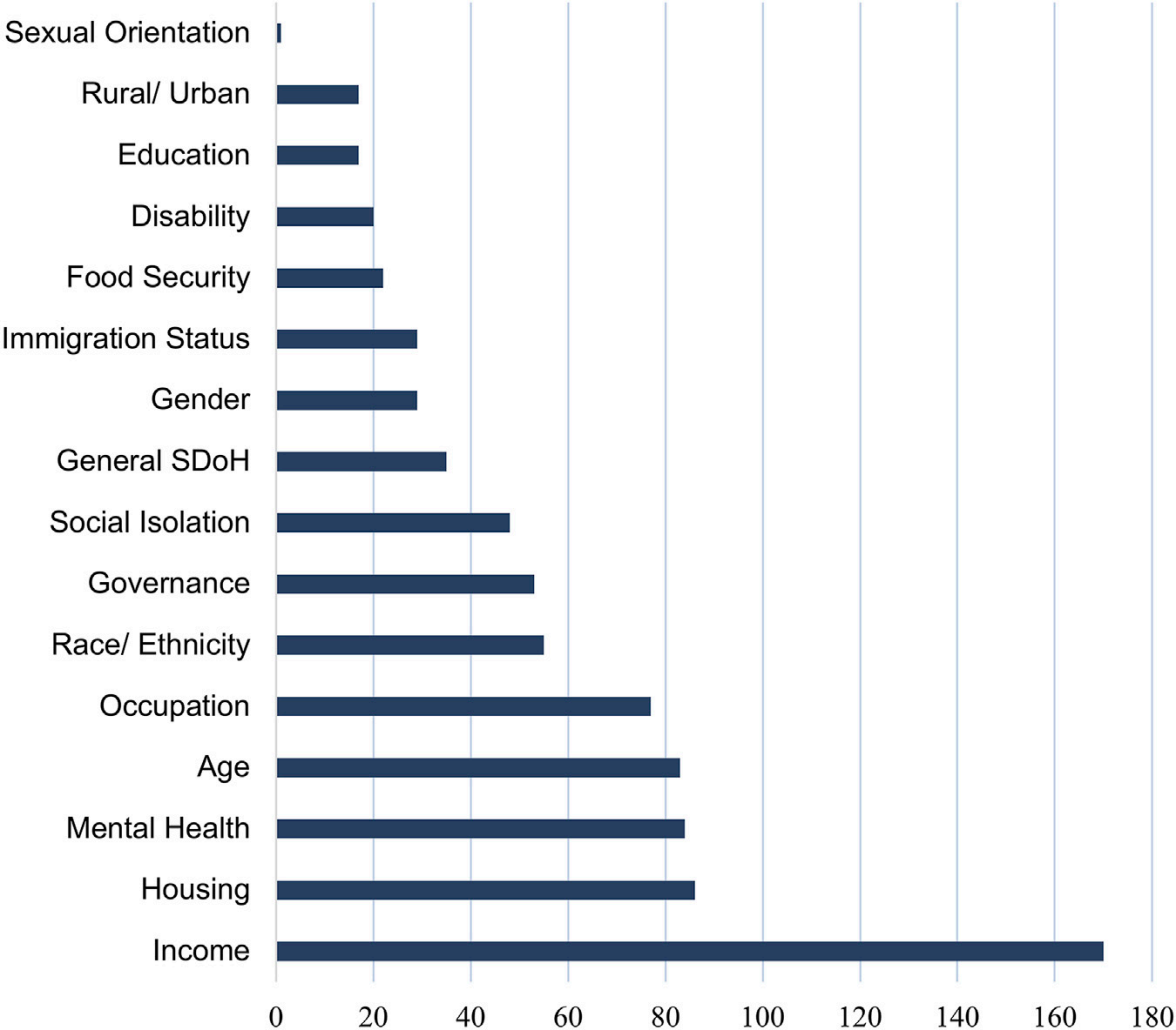
Graph of articles according to social determinant of health discussed (Systematic Review, Global, 2019-2020).

Recommendations addressed all levels of prevention (Table 1) and addressed many social determinants of health. Income, housing, mental health, age and occupation were most discussed in connection to COVID-19 (Figure 2). More specifically, 170 articles spoke about income (including bigger picture discussions of GDP as well as individual income and health insurance). 86 articles spoke about housing, including those experiencing homeless, long-term care residents, and incarcerated individuals. 84 articles discussed the effects having a mental health condition, 83 articles spoke to age (including specific vulnerable populations such as the elderly and children), and 77 articles discussed the interplay between occupation and COVID-19. Other social determinants of health discussed included race and ethnicity, governance, social isolation, gender, immigration, food security, disability, education, geography, and sexual orientation.

**TABLE 1:** Definitions of public health prevention activities by level.

Level of Public Health Prevention	Definition
Primordial prevention	Addressing the foundational risk factors for COVID-19
Primordial prevention: policy	Changing or creating legislation, rules or regulations to protect vulnerable populations
Primordial prevention: research	Increasing knowledge surrounding the social determinants of health and COVID-19
Primordial prevention: advocacy	Calls for mobilization of people in power to protect vulnerable populations
Primary prevention	Preventing infection of COVID-19 through reducing exposure
Primary prevention: telehealth	Delivering healthcare through a virtual communication platform
Primary prevention: communication and education	Improving communication and public education surrounding COVID-19
Primary prevention: quarantine	Preventing the spread of COVID-19 through physical separation
Primary prevention: protective measures	Preventing the spread of infection through personal protective equipment and infection control practices
Primary prevention: unintended consequences of the pandemic/containment	Mitigating negative effects of the COVID-19 pandemic and of enforced containment strategies unrelated to direct COVID-19 infection
Secondary prevention	Detecting and containing COVID-19 in those who are infected
Secondary prevention: COVID-19 testing	Detecting COVID-19 infection
Secondary prevention: contact tracing	Identifying individuals with potential exposure to COVID-19 cases
Secondary prevention: isolation of COVID-19 cases	Preventing the spread of COVID-19 from those who are infected
Tertiary prevention	Mitigating the complications of COVID-19 on those who are infected
Tertiary prevention: supports for COVID-19 patients and contacts	Supporting individuals who are infected or who have had exposure to someone who was infected with COVID-19

### Primordial Prevention

Thirty-seven articles made general recommendations about primordial prevention to address the SDoH of COVID-19. Articles advocated for international cooperation, noting that providing support to low- and middle-income countries (LMIC) will strengthen the global COVID-19 response [19]. Authors recommended addressing longstanding systematic inequalities in income, housing and employment, to minimize social disparities and protect the most vulnerable [20]. Specific calls focused on supporting persons living with HIV [21], immigrants [22], elderly populations [22], homeless populations [22], and those with mental illness [22]. Some articles highlighted the need to include individuals from marginalized populations in the decision making processes [23], so that “their needs and barriers are understood and incorporated into a comprehensive response that works for everyone” [23].

Fifty-two articles proposed specific policy changes to address the SDoH of COVID-19. Many of these articles called for increased funding, for example, to improve health systems [23–25], to increase pandemic preparedness in prisons [26], and to increase access to opioid use disorder treatment programs [27]. Economic support policies for low-income individuals were important given the impact of containment strategies [28], but new policies must be equitable [29]. For example, one article recommended granting “temporary citizenship rights to every person in the country” [30]. Policies must not further disadvantage already marginalized persons [31]. Lau et al. advocated that “restrictions on freedom of movement must not be applied in a discriminatory way to displaced populations” [32]. Fourteen articles suggested new policies or policy changes that would directly assist marginalized populations [33]. These included reexamining judicial policies, like cash bail or pre-trial incarceration, to decrease the prison population [34], protecting the health of migrant workers [35], reducing enforcement of immigration policies that lead to detention [36], allowing homeless individuals to stay in hotel rooms [37], relaxing restrictions on controlled substances prescriptions for individuals with opioid use disorders [27], and increasing elder abuse penalties [38].

Fifty articles recommended COVID-19 research on the SDoH. Some articles advocated for COVID-19 data to be disaggregated by sex/gender [39], race/ethnicity [40], age [41], location [42], and socioeconomic status [43]. Authors called for research into the psychological effects of the pandemic and of containment strategies in general [44], and specific to elderly populations [45], pediatric populations [45], and illicit drug users [46]. Studies discussed the need for developing rapid point-of-care tests for LMIC that may have limited capacity for laboratory testing [47]. Ten articles highlighted the need for research into the impacts of COVID-19 and barriers to care for specific populations, including people who use drugs [48], victims of interpersonal violence [49], elderly populations [50], persons living with HIV [51], those accessing reproductive care [52], and individuals with dementia [53].

Fifteen articles called for health providers and organizations to engage in advocacy. Recommendations were directed at healthcare professionals to use their professional voices to advocate for disadvantaged groups [54–56]. Other articles called for advocacy from international health organizations [57] or governments [58]. Areas for advocacy included continued access to abortion care [59] and mental health treatment [60], increased social supports [61], increased child well-being [62], and increased supports for individuals with substance use disorders [63], who are homeless [55], or who are incarcerated [55].

### Primary Prevention

Many articles focused on improving primary prevention for at-risk populations in order to combat COVID-19. Some recommended that LMIC focus on primary prevention strategies to avoid overburdening healthcare systems with limited capacity [64]. Yamey et al. call for a global resource allocation system “with national allocations determined through a fair and objective process” based on need [65].

Fifty articles suggested using telehealth to reduce COVID-19 exposure. Seven articles focused on extending the scope of, and reducing barriers to, telehealth, such as reducing the restrictions on reimbursement of telehealth services [66], allowing services to cross US State lines [67], and lifting restrictions on prescribing controlled substances [68]. Studies recommended making telehealth services more accessible, including to those without access to adequate technology or internet services [69], those with low technological literacy [70], and individuals with disabilities [71]. Recommendations included making internet access free for the duration of the pandemic [72], providing computers to low income families [67], or delivering services by phone [73]. Seventeen articles recommended telehealth services for mental health care in order to combat the impacts of the pandemic [74], as well as to provide continued care for those with existing mental health conditions [75]. Telehealth may also be used for populations who face barriers accessing care, such as individuals in rural communities [76], requiring stroke care [77], with dementia [78], with chronic health conditions or chronic pain [71], who are homeless [75], who are pregnant [79], with substance use disorders [46], with HIV [46], and those seeking contraceptive or abortion services [23].

Improving communication and education surrounding COVID-19 was discussed in 81 articles. Many articles highlighted specific populations with greater communication and education needs regarding COVID-19 prevention, including homeless populations [80], individuals with substance use disorders [48], residents of LTCs [81], elderly populations [38], and displaced populations [32]. This would require using a variety of media in order to reach a broad audience [82], including social media [83]. Authors focused on the need to ensure that “all public health messaging, technologies, and communications are accessible to all” [84]. This includes providing information in multiple languages [85], including sign languages [85]; in appropriate forms for varying literacy levels [86]; and in ways that are culturally appropriate [87]. Other accessibility recommendations included transparent masks to support those who rely on lip reading [84] and real-time captioning [84]. Articles advocated for increasing communication about the negative consequences of the COVID-19 pandemic not directly related to SARS-CoV-2 infection [88], such as reducing stigma and anti-Asian racism [89], increasing awareness of higher rates of intimate partner violence [90], and mitigating negative impacts on mental health [91].

Thirty-two articles focused on equitable quarantine practices. Six recommended improving quarantine facilities for individuals in unstable or group living situations [37], including reducing crowding in prisons [8], schools [92], and immigrant detention centres [93]. Ivers and Walton state: “we need radical social investments to support the most impoverished, and we must decongest prisons and release detained asylum seekers to prevent unnecessary deaths” [42]. Quarantining homeless populations was discussed, with calls for additional space for proper distancing [94], or for homeless individuals to use empty hotel rooms during quarantine [37]. Authors discussed measures that would allow individuals to stay home to reduce risk of infection [50], including increasing home care for elderly patients [70], remote treatment for individuals with opioid use disorders [95], and mailed prescriptions [96].

Thirty-four articles recommended improving protective measures for at-risk individuals. Some discussed the need for increased personal protective equipment (PPE) for specific groups, including workers with occupational exposure [97], in jails [34], in schools [92], for caregivers of home-care patients [98], in group living facilities [71], in LMIC [99], and in opioid treatment facilities [100]. Some recommended PPE be provided to individuals in high-risk populations, such as elderly [101] and those with disabilities [102], along with increased training on proper use specifically in the context of LTCs [54].

Eighty-two articles included suggestions about the unintended consequences of containment strategies. Articles discussed the consequences of school closures, with recommendations including providing meals to families who normally rely on school-provided meals [103], providing technology to low-income families so children can participate in online learning [92], providing resources for parents now providing full time childcare [92], and providing mental health resources to students who would normally access support at school [45]. Authors aimed to mitigate the increased risk of intimate partner violence and child abuse that accompanies containment strategies, including increased funding and advertising of domestic violence resources [104] and improved monitoring of children and women for signs of violence at home [62]. Some discussed the need to address stigma and discrimination associated with COVID-19 [105], specifically on the basis of race [106] and age [107].

Social isolation is an unintended consequence of containment. Many articles advocated for mental health supports to combat the psychological impacts of containment strategies, including for those with pre-existing mental health conditions [108], pediatric populations [109], healthcare workers [108], individuals with high-exposure occupations [110], and individuals with dementia [111]. Some recommended mitigating isolation through virtual and safe social contact for isolated elderly individuals [112], pediatric populations [113], those with mental health conditions [114], persons with HIV [115], and individuals in rural communities [116].

### Secondary Prevention

Many articles discussed secondary prevention strategies for marginalized populations. Walenski and Del Rio argued that while testing, isolation and contact tracing in underserviced populations is important, it is crucial that this does not exacerbate the marginalization of these communities [20]. Similarly, Quaresima, Naldini and Cirillo suggested secondary prevention strategies should be community-based to ensure that they are understood and respect “local customs and cultural beliefs” [117].

Thirty-four articles advocated for improving COVID-19 testing for marginalized populations. Some commented on the need for fast and accessible testing in resource limited settings in order to control the spread of the virus [118]. Others called for free COVID-19 testing, regardless of citizenship status [20]. Eight articles advocated for increased testing accessibility within specific populations, such as individuals who are homeless [119], in LTCs [120], in immigrant detention facilities [121], in correctional facilities [34], and refugees [32]. Two authors called for wealthier countries to donate testing kits or funds to help control the spread of COVID-19 [122].

Fourteen articles discussed the isolation of COVID-19 cases in marginalized populations. Certain authors recommended providing facilities for isolation in group living settings, such as LTCs [123], immigration detention centres [121], and prisons [8]. Wurcel et al. stated that “there should be dedicated spaces within jails for isolation of persons with confirmed or suspected COVID-19 who are not ill enough to warrant hospital transfer” [34]. Two articles called for improved isolation facilities within hospitals [124]. Zhu et al. discussed protecting healthcare workers [124], and Gupta et al. focused on specialized isolation facilities for psychiatric inpatients [125]. Authors argued that unique support is needed for the isolation of COVID-19 cases in specific populations, including Indigenous peoples [126], homeless populations [127], and individuals with dementia [128].

### Tertiary Prevention

For tertiary prevention, authors recommended creating systems and guidelines to improve care for cases of COVID-19 among vulnerable populations. This includes creating standardized guidelines for assessing and prioritizing patients in resource-limited settings in order to remove biases, such as ageism, from decision-making processes [107], as well as mitigating “additional emotional distress when allocating resources and denying care to patients” [99] on healthcare workers.

Twenty-one articles discussed tertiary prevention recommendations related to increased supports for COVID-19 patients among vulnerable populations. General calls for equitable access to treatment included a recommendation for Medicare for all Americans [97], for Medicaid co-payments to be waived for all Americans [127], for any COVID-19 therapeutics to be made available to all [42], and for any barriers to care to be addressed [129]. Recommendations for disadvantaged groups called for accessible care for international migrant workers [35], the uninsured [85], residents and staff of LTCs [85], incarcerated individuals [85], refugees or ICE detention centers [85], individuals in homeless shelters [85], rural populations [130], individuals who use drugs [48], and displaced populations [32]. Five articles recommended tailoring care to address both COVID-19 and existing comorbidities, including psychiatric illness [54], obesity [131], opioid use disorder [100], spinal cord injury [132], and malnourishment [133]. Articles recommended improving the quality of COVID-19 care for elderly patients through increasing community-based care [134], increasing the scope of care of clinicians in LTCs [135], and allowing visitors for elderly patients nearing death in hospital [136].

## Discussion

We identified 338 commentaries, editorials, and opinion pieces in peer-reviewed publications that offered recommendations on addressing equity and SDoH during the COVID-19 pandemic. Recommendations for primordial prevention focused on improved policy, focused research, and targeted advocacy initiatives. Primary prevention includes accessible telehealth and communication strategies, and equitable quarantine and protective measures. Secondary prevention includes increased testing and more comprehensive isolation practices. Tertiary prevention recommendations advocated for increased supports for marginalized COVID-19 patients. Most articles focused on primordial and primary prevention of COVID-19. Recommendations were consistent, and the SDoH of COVID-19 are a global concern. Almost all SDoH were discussed, with income, housing, mental health, and age most commonly referenced.

Our findings fit with calls to address inequities related to COVID-19 [137, 138]. The US Centers for Disease Control created a health equity strategy for COVID-19, recognizing that the “health impact of COVID-19 has exposed long-standing inequities” [139]. The Pan-American Health Organization, American Medical Association, and Canadian National Collaborating Centre for Determinants of Health have also released responses, considerations, and resources to help inform and build equity-based COVID-19 policy and research [140–142].

Our review had limitations. The body of literature on COVID-19 continues to expand rapidly and our review shows only early articles on this subject. We only reviewed papers published in English. We used a public health prevention framework to organize the article recommendations; there are many other ways these articles could have been coded and analyzed. We attempted to minimize these biases by having multiple reviewers in article selection and extraction stages, as well as by using an established critical appraisal tool. There are further limitations in our methodology of choosing a rapid systemic review, and future work should consider not only a research update but a scoping review to fully analyze this subject.

This is the first synthesis of recommendations on addressing SDoH of COVID-19 and can inform public health strategies and policies. Vulnerable populations should be involved in decision-making processes to create relevant and just policy. Strategies should be adapted for local populations, contexts, and geographies. We know now that COVID-19 is disproportionately impacting individuals according to race, income, occupation, and housing status [7, 143–145]. These impacts can widen inequities in the future. With a third wave already here, the expert recommendations discussed in this rapid review need to move from discussion to implementation in order to lessen the inequitable global impact of COVID-19.
