# Supplementary material for: Reducing Inequities During the COVID-19 Pandemic: A Rapid Review and Synthesis of Public Health Recommendations
Source: Public Health Rev. 2022 Jan 17;42:1604031. doi: 10.3389/phrs.2021.1604031 (PMC8802804; doi:10.3389/phrs.2021.1604031)
Supplement: Supplementary file 1 [file DataSheet1.docx]

**Appendix 1: Search strategy**

Modified from the original search strategy and previously published as an appendix to the following article: Upshaw TL, Brown C, Smith R, Perri M, Ziegler C, et al. (2021) Social determinants of COVID-19 incidence and outcomes: A rapid review. PLOS ONE 16(3): e0248336.<https://doi.org/10.1371/journal.pone.0248336>

**Database: Ovid MEDLINE: Epub Ahead of Print, In-Process & Other Non-Indexed Citations, Ovid MEDLINE® Daily and Ovid MEDLINE® <1946-Present>**

Search Strategy:

--------------------------------------------------------------------------------

1 (nCov or 2019 ncov or novel coronavirus or novel corona virus or covid-19 or SARS-COV-2 or Severe Acute Respiratory Syndrome Coronavirus 2 or coronavirus disease 2019 or coronavirus pandemic or coronavirus epidemic or coronavirus outbreak or corona virus pandemic or corona virus epidemic or corona virus outbreak or corona virus disease 2019 or new coronavirus or new corona virus or new coronaviruses or novel coronaviruses or 2019 ncov or nCov 2019 or SARS Coronavirus 2).tw,kf.

2 (wuhan and (coronavirus or corona virus)).tw,kf.

3 (Severe Acute Respiratory Syndrome Coronavirus 2 or COVID-19).os,ps,rs,ox,px,rx,nm.

4 1 or 2 or 3

5 limit 4 to (english language and yr="2019 -Current")

**Ovid Database: Embase Classic+Embase <1947 to 2020 April 23>**

Search Strategy:

--------------------------------------------------------------------------------

1 (nCov or 2019 ncov or novel coronavirus or novel corona virus or covid-19 or SARS-COV-2 or Severe Acute Respiratory Syndrome Coronavirus 2 or coronavirus disease 2019 or coronavirus pandemic or coronavirus epidemic or coronavirus outbreak or corona virus pandemic or corona virus epidemic or corona virus outbreak or corona virus disease 2019 or new coronavirus or new corona virus or new coronaviruses or novel coronaviruses or 2019 ncov or nCov 2019 or SARS Coronavirus 2).tw.

2 (wuhan and (coronavirus or corona virus)).tw.

3 1 or 2

4 limit 3 to english language

5 limit 4 to yr="2019 -Current"

6 limit 5 to embase

**Ovid Database: APA PsycInfo <1806 to April Week 3 2020>**

Search Strategy:

--------------------------------------------------------------------------------

1 (nCov or 2019 ncov or novel coronavirus or novel corona virus or covid-19 or SARS-COV-2 or Severe Acute Respiratory Syndrome Coronavirus 2 or coronavirus disease 2019 or coronavirus pandemic or coronavirus epidemic or coronavirus outbreak or corona virus pandemic or corona virus epidemic or corona virus outbreak or corona virus disease 2019 or new coronavirus or new corona virus or new coronaviruses or novel coronaviruses or 2019 ncov or nCov 2019 or SARS Coronavirus 2).tw

2 (wuhan and (coronavirus or corona virus)).tw.

3 1 or 2

4 limit 3 to yr="2019 -Current"

**CINAHL Plus**

**Search History**

Interface - EBSCOhost Research Databases
Search Screen - Advanced Search
Database - CINAHL Plus with Full Text

| \| **#** \| **Query** \| **Limiters/Expanders** \| \| --- \| --- \| --- \| \| S5 \| S3 AND S4 \| Search modes - Boolean/Phrase \| \| S4 \| EM 20191201-20200427 \| Search modes - Boolean/Phrase \| \| S3 \| S1 OR S2 \| Limiters - English Language Search modes - Boolean/Phrase \| \| S2 \| (wuhan and (coronavirus or corona virus)) \| Search modes - Boolean/Phrase \| \| S1 \| (nCov or 2019 ncov or novel coronavirus or novel corona virus or covid-19 or SARS-COV-2 or Severe Acute Respiratory Syndrome Coronavirus 2 or coronavirus disease 2019 or coronavirus pandemic or coronavirus epidemic or coronavirus outbreak or corona virus pandemic or corona virus epidemic or corona virus outbreak or corona virus disease 2019 or new coronavirus or new corona virus or new coronaviruses or novel coronaviruses or 2019 ncov or nCov 2019 or SARS Coronavirus 2) \| Search modes - Boolean/Phrase \| |
| --- | --- | --- | --- | --- | --- | --- | --- | --- | --- | --- | --- | --- | --- | --- | --- | --- | --- | --- |

**Cochrane Central Register of Controlled Trials (Wiley)**

(nCov or 2019 ncov or novel coronavirus or novel corona virus or covid-19 or SARS-COV-2 or Severe Acute Respiratory Syndrome Coronavirus 2 or coronavirus disease 2019 or coronavirus pandemic or coronavirus epidemic or coronavirus outbreak or corona virus pandemic or corona virus epidemic or corona virus outbreak or corona virus disease 2019 or new coronavirus or new corona virus or new coronaviruses or novel coronaviruses or 2019 ncov or nCov 2019 or SARS Coronavirus 2) or (wuhan and (coronavirus or corona virus)) in Title Abstract Keyword

**Appendix 2: Data extraction table**

| Reference ID | |
| --- | --- |
| Title | |
| Author(s) | |
| Author(s) Country of Origin | |
| Geographic Context | |
| Article Type | Commentary, Analyses, Editorial, Opinion |
| Social Determinant of health | - Age   - Elderly   - Pediatric - Disability - Education - Food security - General (SDOH in general) - Gender - Governance/Policy   - Healthcare System Capacity - Housing   - Homeless   - Long term care   - Prison - Immigrant/refugee - Income   - Health Insurance   - LMIC - Mental health disorder   - Substance use disorder - Occupation - Race/Ethnicity - Rural/urban - Sexual orientation - Social Isolation |
| Type of Evidence | - Personal opinion/experience/observations related to COVID-19 - Personal opinion/experience/observations related SDOH - Previous research on previous communicable disease outbreaks - Previous research on SDOH - Previous research of other topics - Early studies and reports on COVID-19 |
| Recommendations | - Primordial prevention   - Policy   - Research   - Advocacy - Primary prevention   - Communication and education   - Quarantine   - Protective measures   - Unintended pandemic/containment consequences - Secondary prevention   - COVID-19 testing   - COVID-19 contact tracing   - Isolation of COVID-19 cases - Tertiary prevention   - Support for COVID-19 patients and contacts - Other |
| Notes | |

**Appendix 3: Table of included papers**

| **Author** | **Country** | **Title** | **Journal** | **SDoH** | **Recommendations of Public Health Prevention** | **QA1** | **QA2** | **QA3** | **QA4** | **QA5** | **QA6** |
| --- | --- | --- | --- | --- | --- | --- | --- | --- | --- | --- | --- |
| Abena et al. | Cameroon, South Africa, Belgium, Nigeria, USA, Ghana, Democratic Republic of the Congo, Senegal, Cote d’Ivoire, Rwanda, United Kingdom | Chloroquine and hydroxychloroquine for the prevention or treatment of COVID-19 in Africa: Caution for inappropriate off-label use in healthcare settings | American Journal of Tropical Medicine and Hygiene | income (LMIC), governance/ policy (healthcare system capacity) | Tertiary | + | + | + | + | + | + |
| Acharya | Nepal | Resource poor countries ought to focus on early detection and containment of novel corona virus at the point of entry | Clinical Epidemiology and Global Health | governance/ policy (healthcare system capacity) | Secondary: COVID-19 testing | + | - | - | - | - | - |
| Ademuyiwa et al. | Nigeria, Rwanda, Ethiopia, Malawi, Benin, Togo, Burkina Faso, USA, Uganda, Senegal, United Kingdom, Kenya, Zimbabwe, Ghana | COVID-19 Preparedness Within the Surgical, Obstetric, and Anesthetic Ecosystem in Sub-Saharan Africa | Annals of Surgery | income (LMIC) | Primary: communication and education, protective measures  Tertiary | + | + | + | + | + | + |
| Agoramoorthy | Taiwan | India fights hard to neutralize the spread of Covid-19 | Infection Control and Hospital Epidemiology | income | Primordial: policy | + | ? | - | - | - | - |
| Agyeman,  Laar & Ofori-Asenso | Denmark | Will COVID-19 be a litmus test for post-Ebola sub-Saharan Africa? | Journal of Medical Virology | income (LMIC), housing, governance/ policy (healthcare system capacity), | Primary: communication and education, Quarantine;  Secondary: COVID-19 testing;  Tertiary: Supports | + | + | + | + | + | - |
| Ahmed et al. | UK, USA | Why inequality could spread COVID-19 | The Lancet Public Health | general SDoH, income, occupation, housing | Primordial: policy | + | + | + | + | - | - |
| Ahonsi | Ghana | A research agenda on the sexual and reproductive health dimensions of the COVID-19 pandemic in Africa | African Journal of Reproductive Health | income (LMIC), gender, governance/ policy (healthcare system capacity) | Primordial: research | + | + | + | + | + | + |
| Akiyama,  Spaulding &  Rich | USA | Flattening the curve for incarcerated populations - Covid-19 in jails and prisons | New England Journal of Medicine | housing (prison), race/ ethnicity, housing, mental health disorder, mental health disorder (substance use) | Primary: protective measures  Secondary: isolation  Tertiary: supports | + | + | + | + | + | + |
| Al-Quteimat &  Amer | United Arab Emirates, Jordan | SARS-CoV-2 outbreak: How can pharmacists help? | Research in Social and Administrative Pharmacy | race/ ethnicity, occupation | Primordial: advocacy | + | + | + | + | + | + |
| Alexander et al. | USA | An Epidemic in the Midst of a Pandemic: Opioid Use Disorder and COVID-19 | Annals of Internal Medicine | mental health disorder (substance use) | Primary: protective measures, unintended consequences | + | + | + | + | + | + |
| Altena et al. | France, Germany, Italy, UK, Austria, Sweden | Dealing with sleep problems during home confinement due to the COVID-19 outbreak: Practical recommendations from a task force of the European CBT-I Academy | Journal of Sleep Research | gender, age, mental health disorder, occupation, social isolation | Primary: unintended consequences | + | + | + | + | + | + |
| Andrade | India | COVID-19: Humanitarian and Health Care Crisis in a Third World Country | The Journal of Clinical Psychiatry | income (LMIC), housing, occupation, food security, governance/ policy (healthcare system capacity) | Primordial: policy  Primary: unintended consequences | + | + | + | + | + | + |
| Armitage &  Nellums | UK | COVID-19 and the consequences of isolating the elderly | The Lancet Public Health | age (elderly), social isolation | Primary: unintended consequences | + | + | + | + | + | + |
| Armitage &  Nellums | UK | The COVID-19 response must be disability inclusive | The Lancet Public Health | disability | Primary: communication and education, protective measures | + | + | + | + | + | - |
| Armitage &  Nellums | UK | Considering inequalities in the school closure response to COVID-19 | The Lancet Global Health | age (pediatric), food security, income | Primary: protective measures, unintended consequences | + | + | + | + | + | + |
| Arora at al. | India, USA, Brazil, Italy, Iran, Poland, Germany, Switzerland | Solidarity and transparency against the COVID-19 pandemic | Dermatologic Therapy | income (LMIC) | Primordial: policy | + | + | + | + | + | - |
| Arya &  Gupta | India | COVID-19 outbreak: Challenges for Addiction services in India | Asian Journal of Psychiatry | mental health disorder (substance use), income | Primary: unintended consequences | + | + | + | + | + | ? |
| Asim at al.  Sathian, | Qatar, UK, Nepal | COVID-19 Pandemic: Public Health Implications in Nepal | Nepal Journal of Epidemiology | governance/ policy (healthcare system capacity), housing, education, income | Primordial: advocacy | + | + | + | + | + | + |
| Assari &  Habibzadeh | USA, Iran | The COVID-19 emergency response should include a mental health component | Archives of Iranian Medicine | mental health disorder, age (elderly), occupation, governance policy (healthcare system capacity) | None | + | + | + | + | + | + |
| Auerbach &  Miller | USA | COVID-19 Exposes the Cracks in Our Already Fragile Mental Health System | American Journal of Public Health | mental health disorder, mental health disorder (substance use), housing (LTC) immigrant/ refugee, income (health insurance) | Primordial: policy  Primary: unintended consequences | + | + | + | + | + | + |
| Ayalon | Israel | There is nothing new under the sun: Ageism and intergenerational tension in the age of the COVID-19 outbreak | International Psychogeriatrics | age (elderly) | Primordial: advocacy and policy | + | + | + | + | + | + |
| Ayalon et al. | Israel, Canada, USA, Germany | Aging in Times of the COVID-19 Pandemic: Avoiding Ageism and Fostering Intergenerational Solidarity | The journals of gerontology. Series B, Psychological sciences and social sciences | age (elderly), social isolation | Primary: unintended consequences  Tertiary | + | + | + | + | + | + |
| Azim at al. | Pakistan | COVID-19 as a psychological contagion: A new Pandora's box to close? | Infection Control and Hospital Epidemiology | mental health disorder, race/ ethnicity, income | Primordial: policy  Primary: communication and education | + | + | + | + | + | + |
| Azizy,  Fayaz &  Agirbasli | Afghanistan, Turkey | Do Not Forget Afghanistan in Times of COVID-19: Telemedicine and the Internet of Things to Strengthen Planetary Health Systems | OMICS A Journal of Integrative Biology | income (LMIC), governance/ policy (healthcare system capacity) | Secondary | + | + | + | + | ? | + |
| Bachireddy,  Chen &  Dar | USA | Securing the Safety Net and Protecting Public Health during a Pandemic: Medicaid's Response to COVID-19 | JAMA | income (health insurance), governance/ policy (healthcare system capacity), housing (LTC), housing (homeless) | Primary: communication and education, protective measures  Secondary: COVID-19 testing, isolation  Tertiary: Supports | + | + | + | + | + | - |
| Bajwah et al. | UK, Italy, Germany, Denmark, Australia | Managing the supportive care needs of those affected by COVID-19 | European Respiratory Journal | income | None | + | + | + | + | + | + |
| Baker et al. | New Zealand | New Zealand’s elimination strategy for the COVID-19 pandemic and what is required to make it work | The New Zealand Medical Journal | income, race/ ethnicity | Primordial: policy | + | + | + | + | + | + |
| Balanzá–Martínez et al. | Spain, Canada, Brazil | Lifestyle behaviours during the COVID-19 – time to connect | Acta Psychiatrica Scandinavica | social isolation, mental health disorder | Primordial: research  Primary: communication and education | + | + | - | + | + | - |
| Barazzoni | Italy, Germany, Russia, Croatia, Denmark, Israel | ESPEN expert statements and practical guidance for nutritional management of individuals with SARS-CoV-2 infection | Clinical Nutrition | food security, age (elderly) | Tertiary | + | + | + | + | + | + |
| Bassi &  Hwenda | Switzerland, South Africa | COVID-19: time to plan for prompt universal access to diagnostics and treatments | The Lancet Global Health | income (LMIC) | Primary: protective measures  Tertiary | + | + | + | + | + | - |
| Battegay et al. | Switzerland | 2019-Novel Coronavirus (2019-nCoV): estimating the case fatality rate – a word of caution | Swiss Medical Weekly | general SDoH | None | + | + | + | + | + | + |
| Bayefsky,  Bartz &  Watson | USA | Abortion during the CoviD-19 pandemic - Ensuring access to an essential health service | New England Journal of Medicine | gender | Primordial: advocacy | + | + | + | + | + | + |
| Becker &  Fiellin | USA | When Epidemics Collide: Coronavirus Disease 2019 (COVID-19) and the Opioid Crisis | Annals of Internal Medicine | mental health disorder (substance use) | Primordial: advocacy, policy  Primary: unintended consequences | + | + | + | + | + | + |
| Bell &  Aronoff-Spencer | USA | “Global Health”: Time to refocus while we still have time | American Journal of Tropical Medicine and Hygiene | income (LMIC), governance/ policy (healthcare system capacity) | Primordial: policy | + | ? | + | + | + | + |
| Berger et al. | USA | Covid-19: Control measures must be equitable and inclusive | The BMJ | general SDoH, race/ ethnicity, disability, immigrant/ refugee, income (health insurance) | Primary: communication and education, protective measures  Secondary: COVID-19 testing  Tertiary: supports | + | + | + | + | + | + |
| Bhatia | India | Public engagement is key for containing COVID-19 pandemic | Indian Journal of Medical Research | housing, rural/ urban, immigrant/ refugee | Primary: communication and education | + | + | + | + | + | + |
| Bhopal | UK | COVID-19: Immense necessity and challenges in meeting the needs of minorities, especially asylum seekers and undocumented migrants | Public Health | immigrant/ refugee, race/ ethnicity | Primordial: policy  Primary | + | + | + | + | - | - |
| Boakye, Jenkins & Sharma | USA | Disproportionate impact of COVID-19 pandemic on head and neck cancer survivors | Head and Neck | social isolation, income, rural/ urban, mental health disorder, occupation | Primary: unintended consequences | + | + | + | + | - | - |
| Bong et al. | Singapore, Australia, Zimbabwe, Norway, Canada | The COVID-19 Pandemic: Effects on Low- and Middle-Income Countries | Anesthesia and analgesia | income (LMIC), housing, immigrant/ refugee, governance/ policy (healthcare system capacity), occupation | Primordial: advocacy, policy  Primary: communication and education | + | + | + | + | + | + |
| Bradbury-Jones &  Isham | UK | The pandemic paradox: The consequences of COVID-19 on domestic violence | Journal of Clinical Nursing | gender, age (pediatric) | Primary: unintended consequences | + | + | + | + | + | + |
| Brooke &  Jackson | UK, Australia | Older people and COVID-19: Isolation, risk and ageism | Journal of Clinical Nursing | age (elderly), social isolation, housing (LTC), education | Primary: unintended consequences | + | ? | + | + | + | + |
| Bruns,  Kraguljac &  Bruns | USA | COVID-19: Facts, Cultural Considerations, and Risk of Stigmatization | Journal of Transcultural Nursing | race/ ethnicity, income (LMIC), governance/ policy (healthcare system capacity) | Primary: communication and education | + | + | + | + | + | + |
| Bryant | USA | We Can’t Just Wash Our Hands: a Primary Care Physician’s Thoughts on COVID-19 and Her Community | Journal of General Internal Medicine | occupation | Primary: quarantine | + | + | + | - | - | - |
| Burkle | USA | Political Intrusions into the International Health Regulations Treaty and Its Impact on Management of Rapidly Emerging Zoonotic Pandemics: What History Tells Us | Prehospital and Disaster Medicine | general SDoH | None | + | + | + | + | + | + |
| Burkle | USA | Declining Public Health Protections within Autocratic Regimes: Impact on Global Public Health Security, Infectious Disease Outbreaks, Epidemics, and Pandemics | Prehospital and Disaster Medicine | governance/ policy | Primordial: policy | + | + | + | + | + | + |
| Buss &  Tobar | Brazil | COVID-19 and opportunities for international cooperation in health | Cadernos de Saude Publica | income (LMIC), occupation | Primordial | + | + | + | + | - | ? |
| Butler &  Barrientos | USA | The impact of nutrition on COVID-19 susceptibility and long-term consequences | Brain, Behavior, and Immunity | food security, age (elderly), race/ ethnicity | Secondary | + | + | - | + | + | - |
| Calton,  Abedini &  Fratkin | USA | Telemedicine in the Time of Coronavirus | Journal of Pain and Symptom Management | income | Primordial: advocacy  Primary: telehealth | + | + | + | + | + | + |
| Cappelli &  Cini | Italy | Will the COVID-19 pandemic make us reconsider the relevance of short food supply chains and local productions? | Trends in Food Science and Technology | food security | Primordial: research | + | + | + | + | + | + |
| Carrico et al. | USA | Double Jeopardy: Methamphetamine Use and HIV as Risk Factors for COVID-19 | AIDS and Behavior | sexual orientation, mental health disorder (substance use), race/ ethnicity, mental health disorder | Primordial: research  Primary: protective measures | + | + | + | + | + | + |
| Carta,  Romano &  Orrù | Italy | The True Challenges of the Covid-19 Epidemics: The Need for Essential Levels of Care for All | The Open Respiratory Medicine Journal | income, income (LMIC) | Primordial: policy | + | ? | - | - | - | ? |
| Castillo | USA | Virulent Greed | National Nurse | occupation, income (health insurance) | Primary: protective measures  Tertiary: supports | + | + | + | + | - | - |
| Chatterjee,  Barikar C &  Mukherjee | India | Impact of COVID-19 pandemic on pre-existing mental health problems | Asian Journal of Psychiatry | mental health disorder, social isolation, income | Primordial: policy  Primary: communication and education | + | + | + | + | + | - |
| Chen | USA | Covid-19 and family doctors | Family Medicine | housing (homeless) | None | + | + | + | + | + | + |
| Choo | USA | The Penumbra COVID-19 fault lines | The Lancet | housing, income, race/ ethnicity | None | + | + | + | + | - | ? |
| Chopra &  Sobel | USA | Detroit under Siege: The Enemy Within: The impact of the Covid-19 Collision | Infection Control and Hospital Epidemiology | income, governance/ policy (healthcare system capacity), | None | + | + | + | + | - | - |
| Chung,  Dong &  Li | China | Socioeconomic gradient in health and the covid-19 outbreak | The BMJ | income (LMIC), occupation, income, education, social isolation | Primordial: advocacy  Primary: communication and education | + | + | + | + | + | + |
| Cipriani &  Fiorino | Italy | Access to Care for Dementia Patients Suffering From COVID-19 | American Journal of Geriatric Psychiatry | disability | None | + | + | ? | - | - | - |
| Cluver et al. | UK, South Africa, USA | Parenting in a time of COVID-19 | The Lancet | age (pediatric), housing, income | Primary: unintended consequences | + | + | + | + | + | + |
| Coate | UK | Covid-19 and the rise of racism | The BMJ | race/ ethnicity | Primordial: advocacy  Primary: communication and education | + | + | + | + | + | - |
| Conti et al. | USA, Italy, Greece | How to reduce the likelihood of coronavirus-19 (CoV-19 or SARS-CoV-2) infection and lung inflammation mediated by IL-1 | Journal of Biological Regulators and Homeostatic Agents | income | None | + | + | + | + | + | + |
| Courtet et al. | France | Keep Socially (but Not Physically) Connected and Carry on: Preventing Suicide in the Age of COVID-19 | The Journal of clinical psychiatry | social isolation, income, mental health disorder | Primary: communication and education, quarantine  Tertiary: supports | + | + | + | + | + | - |
| COVID-19 Clinical Research Coalition | Global | Global coalition to accelerate COVID-19 clinical research in resource-limited settings | The Lancet | governance/ policy (healthcare system capacity), income (LMIC) | Primordial: Research | - | ? | + | + | + | - |
| Cowling &  Aiello | Hong Kong, USA | Public Health Measures to Slow Community Spread of Coronavirus Disease 2019 | The Journal of Infectious Diseases | income, occupation | Primary: unintended consequences | + | + | + | + | + | + |
| Cox,  Plavnick &  Brodhead | USA | A Proposed Process for Risk Mitigation During the COVID-19 Pandemic | Behavior Analysis in Practice | occupation | Primary: protective measures  Other | + | + | + | + | + | + |
| Cudjoe &  Abdullah | Hong Kong | Drawing on Kinship Care Support for Older People during a Pandemic (COVID-19): Practice Considerations for Social Workers in Ghana | Journal of Gerontological Social Work | age (elderly), income (LMIC) | Primary: communication and education | + | ? | + | + | - | - |
| Cullen,  Gulati &  Kelly | Ireland | Mental health in the Covid-19 pandemic | QMJ | mental health disorder, occupation | Primary: unintended consequences | + | + | + | + | + | + |
| Das | India | Psychiatrist in post-COVID-19 era – Are we prepared? | Asian Journal of Psychiatry | mental health disorder, age (elderly), occupation, age (pediatric), housing (homeless) | Primordial: research  Primary: unintended consequences | + | + | + | + | + | + |
| Davey et al. | USA, South Africa | Contracting HIV or Contracting SAR-CoV-2 (COVID- 19) in Pregnancy? Balancing the Risks and Benefits | AIDS and Behavior | gender | Primary: unintended consequences | + | + | + | + | + | + |
| Davidson &  Szanton | USA | Nursing homes and COVID-19: we can and should do better | Journal of Clinical Nursing | housing (LTC) | Primordial: policy | + | + | + | + | + | + |
| de Freitas Lima Ventura et al. | Brazil | Challenges of the COVID-19 pandemic: For a Brazilian research agenda in global health and sustainability | Cadernos de Saude Publica | governance/ policy, income (LMIC) | Primordial: research | + | + | ? | + | + | ? |
| De Leo &  Trabucchi | Italy, Australia | The Fight against Covid-19: A Report from the Italian Trenches | International Psychogeriatrics | age (elderly), mental health disorder, housing (LTC), social isolation, occupation | Primary: communication and education | + | + | + | + | - | - |
| DePierro,  Lowe &  Katz | USA | Lessons learned from 9/11: Mental health perspectives on the COVID-19 pandemic | Psychiatry Research | mental health disorder, occupation | Primordial: research  Primary: unintended consequences | + | + | + | + | - | + |
| Devakumar et al. | UK | Racism and discrimination in COVID-19 responses | The Lancet Public Health | race/ ethnicity, income, occupation, immigrant/ refugee | Primordial: policy  Primary: unintended consequences | + | + | + | + | + | + |
| Dietz &  Santos-Burgoa | USA | Obesity and its Implications for COVID-19 Mortality | Obesity | race/ ethnicity | Secondary: COVID-19 testing  Tertiary: supports | + | + | + | + | + | + |
| Dixon | USA | Why Am I, as a Geriatric Medicine Fellow with Symptoms, Unable to Get Tested for COVID-19 While Politicians, Oil Executives, and NBA Players Are? | Journal of the American Geriatrics Society | income | Primordial: advocacy | + | + | + | + | - | - |
| Drain &  Garrett | USA | SARS-CoV-2 pandemic expanding in sub-Saharan Africa: Considerations for COVID-19 in people living with HIV | EClinicalMedicine | income (LMIC) | Primordial: research  Primary: unintended consequences  Secondary: COVID-19 testing | + | + | + | + | + | + |
| Dramé et al. | Martiniques, France, Cameroon, Guadeloupe | Coping with the COVID-19 crisis in Sub-Saharan Africa: let us not leave older people behind! | European Geriatric Medicine | income (LMIC), age (elderly), governance/ policy (healthcare system capacity) | Primary | + | + | + | + | + | - |
| Druss | USA | Addressing the COVID-19 Pandemic in Populations with Serious Mental Illness | JAMA Psychiatry | mental health disorder, housing (homeless), income, income (health insurance), housing (LTC) | Primordial: policy  Primary: communication and education, protective measures | + | + | + | + | + | + |
| Duan &  Zhu | China | Psychological interventions for people affected by the COVID-19 epidemic | The Lancet Psychiatry | mental health disorder, social isolation, income | Primary: unintended consequences | + | + | + | + | + | - |
| Dunn et al. | USA | Feeding low-income children during the CoviD-19 pandemic | New England Journal of Medicine | income, age (pediatric), immigrant/ refugee | Primary: unintended consequences | + | + | + | + | + | - |
| Duong &  Karlawish | USA | Caregiving at a Physical Distance: Initial Thoughts for COVID-19 and Beyond | Journal of the American Geriatrics Society | disability, age (elderly), housing (LTC) | Primary: communication and education, protective measures, unintended consequences | + | + | + | + | + | - |
| Eccleston et al. | UK, USA, Australia, Canada | Managing patients with chronic pain during the COVID-19 outbreak: considerations for the rapid introduction of remotely supported (eHealth) pain management services | Pain | disability, income | Primary: protective measures | + | + | + | + | + | + |
| Eckert | Australia | COVID-19 – Nurses and midwives impact on global security | Australian Journal of Advanced Nursing | income (LMIC), mental health disorder | Primary | + | + | + | + | - | ? |
| EClinicalMedicine | Global | Emerging zoonoses: A one health challenge | EClinicalMedicine | occupation | Primary: Protective measures | - | ? | - | + | - | - |
| Edmonds,  Kneipp &  Campbell | USA | A call to action for public health nurses during the COVID-19 pandemic | Public Health Nursing | gender, income | Primordial: advocacy | + | + | + | + | + | + |
| El-Sadr &  Justman | USA | Africa in the Path of Covid-19 | New England Journal of Medicine | income (LMIC), governance/ policy (healthcare system capacity), housing | Primary: communication and education, unintended consequences  Secondary | + | ? | + | + | + | + |
| Elkind,  Harrington &  Benjamin | USA | The role of the American Heart Association in the global COVID-19 pandemic | Circulation | general SDoH, governance/ policy (healthcare system capacity) | None | + | ? | ? | + | + | - |
| Fakari &  Simbar | Iran | Coronavirus Pandemic and Worries during Pregnancy; a Letter to Editor | Archives of Academic Emergency Medicine | gender | Primary: communication and education, protective measures | + | + | + | + | + | - |
| Fanidi,  Jouven &  Gaye | UK, France | Strategies to control COVID-19 and future pandemics in Africa and around the globe | European heart journal | income (LMIC), food security | Primordial: advocacy  Primary: communication and education  Secondary: COVID-19 testing | + | + | + | + | ? | ? |
| Feng et al. | Hong Kong, USA, UK | Rational use of face masks in the COVID-19 pandemic | The Lancet Respiratory Medicine | age (elderly), race/ ethnicity | Primary: protective measures, unintended consequences | + | + | + | + | + | + |
| Ferrante &  Fearnside | Brazil | Protect Indigenous peoples from COVID-19 | Science | race/ ethnicity | Primary: quarantine  Secondary: isolation | + | + | + | + | + | + |
| Fiorillo &  Gorwood | France | The consequences of the COVID-19 pandemic on mental health and implications for clinical practice | European psychiatry | mental health disorder, occupation | Primordial: advocacy  Primary: unintended consequences | + | + | + | + | + | + |
| Flint,  Bingham &  Iaboni | Canada | Effect of COVID-19 on the Mental Health Care of Older People in Canada | International Psychogeriatrics | age (elderly), mental health disorder, housing (LTC), housing (homeless), social isolation | Primordial: policy  Primary: communication and education, quarantine | + | + | + | + | + | - |
| Galea,  Merchant &  Lurie | USA, Norway | The Mental Health Consequences of COVID-19 and Physical Distancing: The Need for Prevention and Early Intervention | JAMA Internal Medicine | mental health disorder, age, gender, housing (homeless), immigrant/ refugee | Primordial: advocacy, policy | + | ? | + | + | + | + |
| Gandhi,  Yokoe &  Havlir | USA | Asymptomatic Transmission, the Achilles’ Heel of Current Strategies to Control Covid-19 | New England Journal of Medicine | housing (LTC), housing (homeless), housing (prison) | Secondary: COVID-19 testing | + | + | + | + | + | + |
| Garcia &  Duarte | Brazil | Nonpharmaceutical interventions for tackling the COVID-19 epidemic in Brazil | Epidemiologia e servicos de saude : revista do Sistema Unico de Saude do Brasil | general SDoH | None | + | + | + | + | + | + |
| Gardner,  States &  Bagley | Canada, USA | The Coronavirus and the Risks to the Elderly in Long-Term Care | Journal of Aging and Social Policy | housing (LTC), age (elderly), occupation, social isolation | Primary: protective measures, unintended consequences  Secondary: COVID-19 testing, isolation  Tertiary: supports | + | ? | + | + | + | - |
| Garg,  Bhatnagar &  Gangadharan | India | A Case for Participatory Disease Surveillance of the COVID-19 Pandemic in India | JMIR Public Health and Surveillance | education | None | + | + | + | + | + | + |
| Gates | USA | Responding to Covid-19 — A Once-in-a-Century Pandemic? | New England Journal of Medicine | income (LMIC) | Primordial: policy | + | - | + | + | + | - |
| Gaur et al. | USA | Unprecedented solutions for extraordinary times: Helping long-term care settings deal with the COVID-19 pandemic | Infection Control and Hospital Epidemiology | age (elderly) | Primary: protective measures  Secondary: COVID-19 testing, isolation  Tertiary: supports | + | + | + | + | + | - |
| Gausman &  Langer | USA | Sex and Gender Disparities in the COVID-19 Pandemic | Journal of Women's Health | gender, occupation | Primordial: policy | + | ? | + | + | + | + |
| Gautam &  Sharma | India | 2019-nCoV pandemic: A disruptive and stressful atmosphere for Indian academic fraternity | Brain, Behavior, and Immunity | mental health disorder, income | Primary: unintended consequences | + | + | + | + | - | - |
| Ghebreyesus &  Swaminathan | Global (WHO) | Scientists are sprinting to outpace the novel coronavirus | The Lancet | income (LMIC), governance/ policy (healthcare system capacity), age | Secondary: COVID-19 testing, isolation | + | + | + | + | - | - |
| Golberstein,  Wen &  Miller | USA | Coronavirus Disease 2019 (COVID-19) and Mental Health for Children and Adolescents | JAMA Pediatrics | age (pediatric), mental health disorder, income, race/ ethnicity, income (health insurance) | Primordial: policy  Primary: unintended consequences | + | + | + | + | + | + |
| Goldberg | USA | Psychiatry's Niche Role in the COVID-19 Pandemic | The Journal of clinical psychiatry | mental health disorder | None | + | + | + | + | + | - |
| Goldschmidt | USA | The COVID-19 pandemic: Technology use to support the wellbeing of children | Journal of Pediatric Nursing | age (pediatric), social isolation, disability, food security | Primary: unintended consequences | + | + | + | + | + | - |
| Gostin,  Hodge &  Wiley | USA | Presidential Powers and Response to COVID-19 | JAMA | age (elderly), general SDoH | Primary: unintended consequences | + | + | + | + | - | + |
| Graef et al. | USA, Sweden, Canada, France, New Zealand, Germany, Australia, Peru | Festina lente: Hydroxychloroquine, COVID-19 and the role of the rheumatologist | Annals of the Rheumatic Diseases | income, rural/ urban | Primary: unintended consequences | + | + | + | + | + | + |
| Gupta et al. | India | The missing pieces in the jigsaw and need for cohesive research amidst coronavirus infectious disease 2019 global response | Medical Journal Armed Forces India | general SDoH, occupation | Primordial: research  Primary: communication and education, protective measures  Secondary: isolation | + | + | + | + | + | + |
| Gurwitz | USA | COVID-19, Post-acute Care Preparedness and Nursing Homes: Flawed Policy in the Fog of War | Journal of the American Geriatrics Society | housing (LTC), age (elderly) | Primordial: policy  Primary: protective measures  Secondary: COVID-19 testing | + | + | + | + | + | + |
| Halepas &  Ferneini | USA | A Pinch of Prevention is Worth a Pound of Cure: Proactive Dentistry in the Wake of COVID-19 | Journal of Oral and Maxillofacial Surgery | occupation, income, race/ ethnicity | Primary: protective measures | + | + | - | + | - | ? |
| Hall et al. | USA, UK | Centring sexual and reproductive health and justice in the global COVID-19 response | The Lancet | gender, race/ ethnicity, income | Primordial: policy, research | + | + | + | + | + | + |
| Han &  Mosqueda | USA | Elder Abuse in the COVID-19 Era | Journal of the American Geriatrics Society | age (elderly), social isolation | Primordial  Primary: communication and education | + | + | + | + | + | - |
| Hargreaves, J.,  Davey &  Group for lessons from pandemic HIV prevention for the COVID-19 response | UK | Three lessons for the COVID-19 response from pandemic HIV | The Lancet HIV | income (LMIC), income, gender | Primordial: policy, research  Primary: protective measures  Secondary: COVID-19 testing  Other | + | - | + | + | + | ? |
| Hargreaves, S. et al. | UK, Norway, Greece | Europe's migrant containment policies threaten the response to covid-19 | The BMJ | immigrant/ refugee | Primordial: advocacy  Primary: communication and education | + | + | + | + | + | - |
| Heckman et al. | Canada, Switzerland | COVID-19 outbreak measures may indirectly lead to greater burden on hospitals | CMAJ | age (elderly), social isolation | Primary: unintended consequences | + | + | + | + | + | + |
| Hedima,  Adeyemi &  Ikunaiye | Nigeria | Community Pharmacists: On the frontline of health service against COVID-19 in LMICs | Research in Social and Administrative Pharmacy | general SDoH, income (LMIC) | Primary  Secondary: COVID-19 testing | + | + | + | + | + | + |
| Heymann &  Shindo | Global  (WHO) | COVID-19: what is next for public health? | The Lancet | occupation, housing (LTC) | Primary: communication and education | + | + | + | + | + | + |
| Ho, C.,  Chee &  Ho, R. | Singapore | Mental Health Strategies to Combat the Psychological Impact of COVID-19 Beyond Paranoia and Panic | Annals Academy of Medicine Singapore | mental health disorder, occupation, race/ ethnicity | Primary: communication and education, protective measures, unintended consequences  Tertiary: supports | + | + | + | + | + | - |
| Holmes et al. | Sweden, UK, Australia, USA | Multidisciplinary research priorities for the COVID-19 pandemic: a call for action for mental health science | The Lancet Psychiatry | mental health disorder, age | Primordial: research  Primary: unintended consequences | + | + | + | + | + | + |
| Hopman,  Allegranzi &  Mehtar | South Africa, Switzerland, Netherlands | Managing COVID-19 in Low- and Middle-Income Countries | JAMA | income (LMIC), governance/ policy (healthcare system capacity) | Primary: communication and education, protective measures  Secondary: COVID-19 testing | + | + | + | + | + | + |
| Humphreys,  Myint &  Zeanah | USA | Increased Risk for Family Violence During the COVID-19 Pandemic | Pediatrics | age (pediatric), gender, social isolation, occupation | Primordial: advocacy  Primary: unintended consequences | + | + | + | + | + | - |
| Ivers &  Walton, | USA | Novel Coronavirus Disease (COVID-19): Global Health Equity in Pandemic Response | American Journal of Tropical Medicine and Hygiene | housing, income (LMIC), race/ ethnicity, governance/ policy (healthcare system capacity), rural/ urban | Primordial: advocacy, research  Primary: protective measures  Tertiary: supports | + | + | + | + | + | + |
| Jackson et al. | Australia, USA, UK, New Zealand, China | Life in the pandemic: Some reflections on nursing in the context of COVID-19 | Journal of Clinical Nursing | general SDoH, housing (homeless), income, housing (prison), immigrant/ refugee | Primordial: advocacy | + | + | + | - | - | ? |
| James | USA | COVID-19: From Epidemic to Pandemic | Disaster Medicine and Public Health Preparedness | general SDoH | Primordial: advocacy | + | ? | + | + | - | - |
| James | USA | Public Health and COVID-19: From Response to Recovery | Disaster Medicine and Public Health Preparedness | income, age (elderly) | Primary: communication and education, protective measures  Other | + | - | + | + | - | - |
| Jawaid | Switzerland | Protecting older adults during social distancing | Science | social isolation | Other | + | + | + | + | + | + |
| Jenkins et al. | USA | COVID-19 During the Opioid Epidemic – Exacerbation of Stigma and Vulnerabilities | Journal of Rural Health | mental health disorder (substance use), governance/ policy (healthcare system capacity), education, income | Primordial: research  Primary: communication and education, protective measures, unintended consequences  Tertiary: supports | + | + | + | + | + | + |
| John et al. | USA | Lessons Never Learned: Crisis and gender-based violence | Developing World Bioethics | gender | Primordial: advocacy, policy, research  Primary: unintended consequences | + | + | + | + | + | + |
| Jones | USA | History in a Crisis — Lessons for Covid-19 | New England Journal of Medicine | general SDoH, race/ ethnicity, occupation | None | + | + | + | + | + | + |
| Joob &  Wiwanitkit | India, Thailand | COVID-19, School Closings, and Weight Gain | Obesity | age (pediatric), income (LMIC), food security | None | + | + | + | + | + | + |
| Jung & Jun | Republic of Korea, USA | Mental health and psychological intervention amid COVID-19 outbreak: Perspectives from South Korea | Yonsei Medical Journal | race/ ethnicity | Primordial: advocacy | + | + | + | + | + | - |
| Kabir et al. | Pakistan | COVID-19 pandemic and economic cost; impact on forcibly displaced people | Travel Medicine and Infectious Disease | immigrant/ refugee | Primordial: advocacy | + | + | + | + | + | - |
| Kaebnick | USA | All People | Hastings Center Report | general SDoH | None | ? | ? | ? | / | - | / |
| Kakol,  Upson &  Sood | USA | Susceptibility of Southwestern American Indian Tribes to Coronavirus Disease 2019 (COVID-19) | Journal of Rural Health | race/ ethnicity, general SDoH | Primordial: policy, research  Primary | + | + | + | + | + | + |
| Kamath, S.,  Kamath, R. &  Salins | India | COVID-19 pandemic in India: Challenges and silver linings | Postgraduate Medical Journal | income (LMIC), rural/ urban, governance/ policy (healthcare system capacity), occupation | Primordial: research  Secondary: COVID-19 testing | + | + | + | + | - | + |
| Kapata et al. | UK, Zambia, Nigeria, Italy, Congo | Is Africa prepared for tackling the COVID-19 (SARS-CoV-2) epidemic. Lessons from past outbreaks, ongoing pan-African public health efforts, and implications for the future | International Journal of Infectious Diseases | income (LMIC) | Primordial: policy | + | + | + | + | + | + |
| Kar, P. | UK | Partha Kar: What will come after the covid-19 crisis? | The BMJ | race/ ethnicity, income | None | + | + | + | + | - | + |
| Kar, S. et al. | India, Bangladesh, Indonesia, United Kingdom | Homeless mentally ill people and COVID-19 pandemic: The two-way sword for LMICs | Asian Journal of Psychiatry | housing (homeless), mental health disorder, income (LMIC) | Primary: communication and education  Secondary: COVID-19 testing | + | + | - | + | - | - |
| Kar, S. et al. | India, Bangladesh, Nepal, Indonesia, United Kingdom | COVID-19 pandemic and addiction: Current problems and future concerns | Asian Journal of Psychiatry | mental health disorder (substance use), social isolation | Primary: unintended consequences | + | + | ? | + | - | + |
| Karnon | Australia | The Case for a Temporary COVID-19 Income Tax Levy Now, During the Crisis | Applied Health Economics and Health Policy | income, occupation, general SDoH | Primordial: policy | + | + | ? | + | + | - |
| Kaufman et al. | USA, UK, Germany | A global needs assessment in times of a global crisis: world psychiatry response to the COVID-19 pandemic | BJPsych Open | mental health disorder, general SDoH | Primordial: research  Primary: unintended consequences | + | + | + | + | + | + |
| Kavoor | Australia | COVID-19 in People with Mental Illness: Challenges and Vulnerabilities | Asian Journal of Psychiatry | mental health disorder, housing | Primary: unintended consequences | + | + | + | + | + | + |
| Keller &  Wagner, | USA | COVID-19 and immigration detention in the USA: time to act | The Lancet Public Health | immigrant/ refugee, rural/ urban | Primary: protective measures | + | + | + | + | + | - |
| Kelly | Ireland | Covid-19 (Coronavirus): Challenges for Psychiatry | The British Journal of Psychiatry | mental health disorder, housing (homeless), disability, housing (prison), income | Primary: unintended consequences | + | + | + | + | + | - |
| Khadka,  Hashmi &  Usman | Nepal, Pakistan | Preventing COVID-19 in low- and middle-income countries | Drugs and Therapy Perspectives | income (LMIC) | Primary: communication and education, protective measures | + | + | + | + | + | - |
| Khan et al. | China | Novel coronavirus, poor quarantine, and the risk of pandemic | Journal of Hospital Infection | income (LMIC), governance/ police (healthcare system capacity) | Primary: quarantine | + | + | + | + | + | - |
| Khoo &  Lantos | Malaysia, USA | Lessons learned from the COVID-19 pandemic | Acta Paediatrica, International Journal of Paediatrics | income, race/ ethnicity, age (pediatric), occupation | Primordial: advocacy  Primary: unintended consequences | + | + | + | + | + | + |
| Khosravi | Iran | Perceived risk of COVID-19 pandemic: The role of public worry and trust | Electronic Journal of General Medicine | general SDoH, education, income | Primary: communication and education | + | + | + | + | + | + |
| Khoury &  Karam | Lebanon | Impact of COVID-19 on Mental Healthcare of Older Adults: Insights from Lebanon (Middle East) | International Psychogeriatrics | age (elderly), income 9LMIC), mental health disorder, food security, income (health insurance) | Primordial: policy  Primary: communication and education | + | + | + | + | + | - |
| Khunti et al. | UK | Is ethnicity linked to incidence or outcomes of covid-19? | The BMJ | race/ ethnicity, housing, income, occupation | Primordial: research | + | + | + | + | + | - |
| Kickbusch &  Leung | Switzerland, China | Response to the emerging novel coronavirus outbreak | The BMJ | income (LMIC), race/ ethnicity | Primordial: policy | + | + | + | + | - | + |
| Kidd | Australia | Australia’s primary care COVID-19 response | Australian journal of general practice | race/ethnicity, age (elderly) | None | + | + | + | + | + | - |
| Kim, S.W. &  Su | Republic of Korea | Using psychoneuroimmunity against COVID-19 | Brain, Behavior, and Immunity | mental health disorder, occupation | None | + | + | + | + | + | + |
| Kim, S.Y.H. & Grady | USA | Ethics in the time of COVID: What remains the same and what is different | Neurology | disability, housing, age (elderly) | Primordial: advocacy | + | + | + | + | + | + |
| Kinner et al. | Australia, Canada, Spain, UK | Prisons and custodial settings are part of a comprehensive response to COVID-19 | The Lancet Public Health | housing (prison) | Primordial: advocacy  Primary: communication and education, quarantine, protective measures  Secondary: isolation | + | + | + | + | + | - |
| Kluge et al. | Denmark, Switzerland | Refugee and migrant health in the COVID-19 response | The Lancet | immigrant/ refugee, housing | Primordial: advocacy  Primary: protective measures | + | + | + | + | + | + |
| Koh | Singapore | Occupational risks for COVID-19 infection | Occupational Medicine | occupation | Primary: protective measures, unintended consequences | + | + | + | + | + | + |
| Kong | USA | What COVID-19 means for non-neurotypical children and their families | Pediatric Research | disability, social isolation | Primary: communication and education, unintended consequences | + | ? | + | + | - | - |
| Kotecha | Australia | Challenges posed by COVID-19 to children with cancer | The Lancet Oncology | age (pediatric), income (LMIC) | Primary: protective measures | + | + | + | + | - | - |
| Kretchy,  Asiedu-Danso &  Kretchy | Ghana | Medication management and adherence during the COVID-19 pandemic: Perspectives and experiences from low-and middle-income countries | Research in Social and Administrative Pharmacy | income (LMIC), governance/ policy (healthcare system capacity) | Primary: unintended consequences | + | + | + | + | + | + |
| Kuwahara,  Kuroda &  Fukuda | Japan | COVID-19: Active measures to support community-dwelling older adults | Travel Medicine and Infectious Disease | age (elderly), social isolation | Primary: unintended consequences | + | + | + | + | + | - |
| LaCourse,  John-Stewart & Waldorf, | USA, Sweden | Importance of inclusion of pregnant and breastfeeding women in COVID-19 therapeutic trials | Clinical Infectious Diseases | gender | Primordial: research | + | + | + | + | + | + |
| Lai, C. et al. | Taiwan | COVID-19 in long-term care facilities: An upcoming threat that cannot be ignored | Journal of Microbiology, Immunology and Infection | housing (LTC) | Primary  Secondary | + | + | + | + | + | + |
| Lai, Y.,  Yeung &  Celi | USA, Singapore | Urban Intelligence for Pandemic Response: Viewpoint | JMIR Public Health and Surveillance | rural/ urban, general SDoH | Primordial: policy | + | + | + | + | + | + |
| Lau et al. | USA | COVID-19 in humanitarian settings and lessons learned from past epidemics | Nature Medicine | immigrant/ refugee | Primordial: policy  Primary: communication and education  Secondary: COVID-19 testing  Tertiary: supports | + | + | + | + | + | + |
| Laupacis | Canada | Working together to contain and manage COVID-19 | CMAJ | occupation, housing (homeless), mental health disorder, race/ ethnicity, income | Primary: protective measures, unintended consequences | + | + | + | - | - | - |
| Lee | UK | Wuhan novel coronavirus (COVID-19): why global control is challenging? | Public Health | income (LMIC) | Other | + | + | + | + | + | - |
| Liem et al. | China, USA | The neglected health of international migrant workers in the COVID-19 epidemic | The Lancet Psychiatry | occupation, mental health disorder, income (health insurance) | Primordial: policy  Primary: communication and education  Tertiary: supports | + | + | + | + | + | + |
| Lima, C.K.T. et al. | Brazil | The emotional impact of Coronavirus 2019-nCoV (new Coronavirus disease) | Psychiatry Research | age (elderly, occupation, mental health disorder) | None | + | + | + | + | + | - |
| Lima, N.N.R. et al. | Brazil | People experiencing homelessness: Their potential exposure to COVID-19 | Psychiatry Research | housing, income, mental health disorder | Primordial: advocacy | + | + | + | - | - | - |
| Litewka & Heitman | USA | Latin American healthcare systems in times of pandemic | Developing World Bioethics | income (LMIC), food security, income, governance/ policy (healthcare system capacity) education | None | + | + | + | + | + | + |
| Liu, J.J. et al. | China | Mental health considerations for children quarantined because of COVID-19 | The Lancet Child & Adolescent Health | age (pediatric) | Primary: unintended consequences | + | + | + | + | + | - |
| Liu, X. | China | Containing COVID-19 in rural and remote areas: experiences from China | Journal of Travel Medicine | rural/ urban, governance/ policy (healthcare system capacity), occupation, income | Primary: communication and education, unintended consequences | + | ? | + | + | - | - |
| Lloyd-Sherlock et al. | UK, Brazil, South Africa | WHO must prioritise the needs of older people in its response to the covid-19 pandemic | The BMJ | age (elderly), housing (LTC) | Primordial: advocacy  Primary: communication and education | + | + | + | - | - | - |
| Lloyd-Sherlock et al. | UK, South Africa | Bearing the brunt of covid-19: Older people in low and middle income countries | The BMJ | income (LMIC), social isolation, housing (LTC), governance/ policy (healthcare system capacity), age (elderly) | Primordial: advocacy | + | + | + | + | + | - |
| Logie &  Turan | Canada, USA | How Do We Balance Tensions Between COVID-19 Public Health Responses and Stigma Mitigation? Learning from HIV Research | AIDS and Behavior | race/ ethnicity, housing (homeless), housing (prison), immigrant/ refugee, gender | Primary: communication and education | + | + | + | + | + | + |
| Lopez &  Holmes | USA | Raids on Immigrant Communities During the Pandemic Threaten the Country's Public Health | American Journal of Public Health | immigrant/ refugee | Primordial: policy | + | + | + | + | + | + |
| Louis-Jean et al. | USA | Coronavirus (COVID-19) in Haiti: A Call for Action | Journal of Community Health | income (LMIC), governance/ policy (healthcare system capacity) | Primary: communication and education | + | ? | + | + | + | - |
| Loveday | UK | Fear, explanation and action – the psychosocial response to emerging infections | Journal of Infection Prevention | education, age (elderly), race/ ethnicity, disability, immigrant/ refugee | Primary: communication and education | + | ? | + | + | + | - |
| Lucchese &  Pianta | Italy | The Coming Coronavirus Crisis: What Can We Learn? | Intereconomics | general SDoH, mental health disorder (substance use), mental health disorder, income | Primordial: policy | + | ? | - | + | + | - |
| Lyon | USA | Covid-19, cancer, and financial toxicity | Oncology Nursing Forum | occupation, income, housing (homeless) | Primordial: advocacy, research | + | + | + | + | + | - |
| Mahmood et al. | Bangladesh, USA | Global Preparedness Against COVID-19: We Must Leverage the Power of Digital Health | JMIR Public Health and Surveillance | income (LMIC), education, social isolation | Primary: communication and education, quarantine  Secondary: COVID-19 testing | + | + | - | + | + | + |
| Malta,  Rimoin &  Strathdee | Canada, USA, Brazil | The coronavirus 2019-nCoV epidemic: Is hindsight 20/20? | EClinicalMedicine | occupation, race/ ethnicity | Primary: communication and education | + | + | + | + | + | - |
| Mamun &  Griffiths | Bangladesh, UK | First COVID-19 suicide case in Bangladesh due to fear of COVID-19 and xenophobia: Possible suicide prevention strategies | Asian Journal of Psychiatry | age (elderly), education, mental health disorder | Primary: unintended consequences | + | + | + | + | + | + |
| Manderson &  Levine | South Africa, Australia | COVID-19, Risk, Fear, and Fall-out | Medical Anthropology | income (LMIC), housing, immigrant/ refugee, occupation, income | None | + | + | + | + | + | + |
| Mann et al. | UK, USA | Athletes as community; Athletes in community: Covid-19, sporting mega-events and athlete health protection | British Journal of Sports Medicine | occupation | None | + | + | + | + | + | - |
| Mannix,  Lee &  Fleegler | USA | Coronavirus Disease 2019 (COVID-19) and Firearms in the United States: Will an Epidemic of Suicide Follow? | Annals of internal medicine | mental health disorder, income, social isolation | Primordial: policy  Primary | + | + | + | + | + | + |
| Markus &  Brainin | UK, Austria | COVID-19 and stroke—A global World Stroke Organization perspective | International Journal of Stroke | income (LMIC), governance/ policy (healthcare system capacity), race/ ethnicity | Primary: communication and education | + | + | + | + | + | + |
| Marsden et al. | UK, Australia, USA | Mitigating and learning from the impact of COVID-19 infection on addictive disorders | Addiction | mental health disorder (substance use), mental health disorder | Primordial: research | + | ? | + | + | + | - |
| Marziali et al. | Canada, USA | Physical Distancing in COVID-19 May Exacerbate Experiences of Social Isolation among People Living with HIV | AIDS and Behavior | social isolation, age (elderly  0 | Primary: unintended consequences | + | + | + | + | + | + |
| Mashamba-Thompson &  Crayton | South Africa, USA | Blockchain and Artificial Intelligence Technology for Novel Coronavirus Disease 2019 Self-Testing | Diagnostics | income (LMIC), governance/ policy (healthcare system capacity), rural/ urban | Secondary: COVID-19 testing | + | + | + | + | + | + |
| Mazumder,  Hossain &  Das | Bangladesh, USA | Geriatric Care during Public Health Emergencies: Lessons Learned from Novel Corona Virus Disease (COVID-19) Pandemic | Journal of Gerontological Social Work | age (elderly) | Primordial: policy  Primary: protective measures  Tertiary: supports | + | + | + | + | - | - |
| McCartney | UK | Medicine: before COVID-19, and after | The Lancet | income | None | + | + | + | + | - | - |
| McKee &  Stuckler | UK, Italy | If the world fails to protect the economy, COVID-19 will damage health not just now but also in the future | Nature Medicine | general SDoH, income, mental health disorder, immigrant/ refugee, housing | Primordial: advocacy | + | + | + | + | + | + |
| Merchant &  Lurie | USA | Social Media and Emergency Preparedness in Response to Novel Coronavirus | JAMA | general SDoH, age (elderly), income, disability, housing | Primary: communication and education, unintended consequences | + | + | + | + | + | + |
| Meyer et al. | USA, Mexico | COVID-19 and the coming epidemic in US immigration detention centres | The Lancet Infectious Diseases | immigrant/ refugee, housing (prison) rural/ urban | Primordial: policy  Secondary: COVID-19 testing, isolation | + | + | + | + | + | + |
| Mobasheri | Finland, Lithuania, Netherlands, United Kingdom | COVID-19, osteoarthritis and women's health | Case Reports in Women's Health | gender, social isolation | Primary: unintended consequences | + | + | + | + | + | + |
| Moesmann Madsen,  Dines &  Hieronymus | Denmark | Optimizing psychiatric care during the COVID-19 pandemic | Acta Psychiatrica Scandinavica | mental health disorder | Primordial: research  Primary: communication and education | + | + | + | + | + | + |
| Mohamed et al. | Iran, Bahrain, Venezuela, USA, Finland, Bulgaria, Lebanon, Afghanistan, Zimbabwe, Bangladesh, Egypt, Indonesia, Sudan, Nigeria, Austria, Armenia, Malaysia, Hungary, Tunis, Germany, Turkey, Netherlands, Italy | Borderless collaboration is needed for COVID-19; A disease that knows no borders | Infection Control and Hospital Epidemiology | income (LMIC) | Secondary: COVID-19 testing | + | + | - | - | + | - |
| Mol &  Caldas | Brazil | Can the human coronavirus epidemic also spread through solid waste? | Waste Management and Research | occupation, income (LMIC) | Primary: protective measures | + | ? | + | + | + | - |
| Momplaisir | USA | The COVID-19 Pandemic: We Are All in This Together | Clinical Infectious Diseases | race/ ethnicity, income (LMIC) | None | + | + | + | + | + | + |
| Monjur &  Hassan | Australia, Bangladesh | Early phases of COVID-19 management in a low-income country: Case of Bangladesh | Infection Control and Hospital Epidemiology | income (LMIC), governance/ policy (healthcare system capacity, occupation | Primary | + | + | + | - | - | - |
| Monteith et al. | USA | Preventing Suicide in Rural Communities During the COVID-19 Pandemic | Journal of Rural Health | rural/ urban, social isolation | Primary: unintended consequences | + | + | + | + | + | + |
| Morley &  Vellas | USA, France | COVID-19 and Older Adult | Journal of Nutrition, Health and Aging | age (elderly), social isolation | Secondary: isolation | + | + | + | + | + | ? |
| Mukhtar | Pakistan | Preparedness and proactive infection control measures of Pakistan during COVID-19 pandemic outbreak | Research in Social and Administrative Pharmacy | mental health disorder, race/ ethnicity | Primordial: research | + | ? | + | + | - | - |
| Munthali &  Xuelian | Malawi, China | Covid-19 outbreak on Malawi perspective | Electronic Journal of General Medicine | income (LMIC) | Primary: communication and education, quarantine  Secondary: COVID-19 testing | + | ? | + | ? | + | - |
| Murphy et al. | UK, Iran | Economic sanctions and Iran's capacity to respond to COVID-19 | The Lancet Public Health | governance/ policy (healthcare system capacity), income (LMIC) | Primordial: policy | + | + | + | + | - | - |
| Musa et al. | China, Sudan, Pakistan | Global outbreak of COVID-19: A new challenge? | Journal of Infection in Developing Countries | income (LMIC) | Primary: unintended consequences | + | + | + | - | - | - |
| Nachega,  Seydi &  Zumla | UK, USA, Senegal, South Africa | The Late Arrival of COVID-19 in Africa - Mitigating Pan-Continental Spread | Clinical infectious diseases : an official publication of the Infectious Diseases Society of America | governance/ policy (healthcare system capacity) | Primary: communication and education, protective measures | + | + | + | + | + | + |
| Naja &  Hamadeh | Lebanon | Nutrition amid the COVID-19 pandemic: a multi-level framework for action | European Journal of Clinical Nutrition | age (elderly) | Primary: unintended consequences | + | + | + | + | + | + |
| Nanda et al. | USA | Contraception in the Era of COVID-19 | Global health, science and practice | gender, income (LMIC) | Primary: unintended consequences | + | + | + | + | + | - |
| Napoli &  Nioi | Italy | Global Spread of Coronavirus Disease 2019 and Malaria: An Epidemiological Paradox in the Early Stage of A Pandemic | Journal of Clinical Medicine | income (LMIC) | None | + | ? | ? | + | + | + |
| Nature Cancer | Global | On being human in the face of a pandemic | Nature Cancer | general SDoH, housing, occupation, income (LMIC) | Primordial: advocacy | - | ? | + | + | + | + |
| Neto et al. | Brazil | When basic supplies are missing, what to do? Specific demands of the local street population in times of coronavirus – a concern of social psychiatry | Psychiatry Research | housing (homeless), mental health disorder, mental health disorder (substance use), income, food security | Primordial: advocacy, policy  Primary: communication and education | + | + | + | + | - | - |
| Ng | UK | The pandemic of hate is giving COVID-19 a helping hand | American Journal of Tropical Medicine and Hygiene | race/ ethnicity | Primordial: advocacy | + | + | + | + | + | + |
| Nicol et al. | USA, Canada | Action at a Distance: Geriatric Research during a Pandemic | Journal of the American Geriatrics Society | age (elderly), social isolation | Primordial: research  Primary: communication and education, protective measures, unintended consequences | + | + | ? | + | + | + |
| Nicol et al. | USA | "What Were You Before the War?" Repurposing Psychiatry During the COVID-19 Pandemic | The Journal of Clinical Psychiatry | mental health disorder, age, social isolation | Primary: unintended consequences | + | + | + | + | + | + |
| Nicola et al. | UK | The socio-economic implications of the coronavirus pandemic (COVID-19): A review | International Journal of Surgery | income, food security, social isolation, education, occupation | Primordial: policy  Secondary: COVID-19 testing | + | + | + | + | + | + |
| Nkengasong & Mankoula | Ethiopia | Looming threat of COVID-19 infection in Africa: act collectively, and fast | The Lancet | governance/ policy (healthcare system capacity), income (LMIC) | Primordial: policy  Primary: quarantine  Tertiary: supports | + | + | + | + | + | + |
| Nuwagira &  Muzoora | Uganda | Is Sub-Saharan Africa prepared for COVID-19? | Tropical Medicine and Health | income (LMIC), governance/ policy (healthcare system capacity) | Primordial: policy | + | + | + | + | + | + |
| O’Neill | Ireland | Protecting Our Longevity Dividend During Covid-19 | Irish Medical Journal | age (elderly), housing (LTC), disability | Primordial: policy  Primary: unintended consequences  Tertiary: supports | + | + | + | + | + | - |
| Oliveira,  Abranches &  Lana | Brazil | Food (in)security in Brazil in the context of the SARS-CoV-2 pandemic | Cadernos de saude publica | food security, occupation, housing (homeless) | Primary: unintended consequences | + | + | + | + | + | - |
| Ornell et al. | Brazil | "Pandemic fear” and COVID-19: mental health burden and strategies | Brazilian Journal of Psychiatry | mental health disorder | Primary: unintended consequences | + | + | + | + | + | - |
| Paakkari &  Okan | Germany | COVID-19: health literacy is an underestimated problem | The Lancet Public Health | education | None | + | + | - | ? | + | + |
| Page et al. | USA | Undocumented U.S. Immigrants and COVID-19 | New England Journal of Medicine | immigrant/ refugee, income (health insurance) | Primary: protective measures, unintended consequences  Tertiary: supports | + | + | + | + | + | + |
| Paintsil | USA | COVID-19 threatens health systems in sub-Saharan Africa: The eye of the crocodile | Journal of Clinical Investigation | governance/ policy (healthcare system capacity), income (LMIC) | Primordial: research, policy  Primary: communication and education, quarantine | + | + | + | + | + | + |
| Palipana | Australia | COVID-19 and spinal cord injuries: The viewpoint from an emergency department resident with quadriplegia | EMA - Emergency Medicine Australasia | disability, rural/ urban | Primary: quarantine  Tertiary | + | + | + | + | + | + |
| Park, S.C. &  Park, Y.C. | Republic of Korea | Mental health care measures in response to the 2019 novel coronavirus outbreak in Korea | Psychiatry Investigation | occupation, mental health disorder | Primordial: research | + | + | + | + | + | + |
| Parmet &  Sinha | USA | Covid-19 - The law and limits of quarantine | New England Journal of Medicine | occupation, immigrant/ refugee, income (health insurance) | Primordial: advocacy  Primary: unintended consequences  Secondary: COVID-19 testing  Tertiary: supports | + | + | + | + | + | + |
| Patel et al. | USA | Ethical and Legal Challenges During the COVID-19 Pandemic – Are We Thinking About Rural Hospitals? | Journal of Rural Health | rural/ urban, age (elderly) | Primordial: advocacy and policy | + | + | + | ? | + | ? |
| Patrick,  Stanbrook &  Laupacis | Canada | Social distancing to combat COVID-19: We are all on the front line | CMAJ | housing, occupation | Primordial: advocacy, policy  Primary: communication and education | + | + | + | + | + | + |
| Peate | UK | Self-isolation and the homeless population | The British Journal of Nursing | housing (homeless) | Primary: quarantine | + | + | ? | - | - | - |
| Pfefferbaum & North | USA | Mental Health and the Covid-19 Pandemic | New England Journal of Medicine | mental health disorder, mental health disorder (substance use), age (elderly), housing, income | Primary: unintended consequences | + | + | + | + | + | + |
| Pineda &  Corburn | USA | Disability, Urban Health Equity, and the Coronavirus Pandemic: Promoting Cities for All | Journal of Urban Health | disability, income | Primordial: policy  Primary: communication and education, unintended consequences | + | + | + | + | + | + |
| Poole et al. | USA | Responding to the COVID-19 pandemic in complex humanitarian crises | International Journal for Equity in Health | governance/ policy (healthcare system capacity), governance/ policy, housing, food security, gender | Primordial: policy  Primary: communication and education | + | + | + | + | + | + |
| Prusaczyk | USA | Strategies for Disseminating and Implementing COVID-19 Public Health Prevention Practices in Rural Areas | Journal of Rural Health | rural/ urban, age (elderly) | Primary: communication and education, protective measures, unintended consequences | + | + | + | + | + | + |
| Quaresima,  Naldini &  Cirillo | Italy | The prospects for the SARS ‐CoV‐2 pandemic in Africa | EMBO Molecular Medicine | income (LMIC), income | Primordial: research  Secondary  Other | + | + | + | + | - | - |
| Rahimi &  Talebi Bezmin Abadi | Australia, Iran | Transparency and information sharing could help abate the COVID-19 pandemic | Infection Control and Hospital Epidemiology | income (LMIC) | Primary: communication and education | + | + | + | + | ? | ? |
| Rathi et al. | India | Hydroxychloroquine prophylaxis for COVID-19 contacts in India | The Lancet Infectious Diseases | income (LMIC) | Primary: unintended consequences | + | + | + | + | + | + |
| Ratner et al. | USA | Reflections on resilience during the COVID-19 pandemic: Six lessons from working in resource-denied settings | American Journal of Tropical Medicine and Hygiene | income (LMIC), race/ ethnicity | Primordial: advocacy  Primary: unintended consequences | + | + | + | + | + | ? |
| Ribas et al. | Brazil | Coronavirus Disease 2019 (COVID-19) and healthcare-associated infections: Emerging and future challenges for public health in Brazil | Travel Medicine and Infectious Disease | income (LMIC), governance/ policy (healthcare system capacity) | None | + | + | ? | - | + | + |
| Rollins | USA | The Coronavirus: Exposing Our Nation’s Vulnerabilities | Pediatric Nursing | age (pediatric), income, food security, occupation | Primordial | + | ? | + | - | - | - |
| Rosenthal et al. | UK | Impacts of COVID-19 on vulnerable children in temporary accommodation in the UK | The Lancet Public Health | age (pediatric), housing, gender | Primary: protective measures | + | + | + | + | + | - |
| Rundle et al. | USA | COVID-19–Related School Closings and Risk of Weight Gain Among Children | Obesity | age (pediatric), race/ ethnicity, food security | Primary: unintended consequences | + | ? | + | + | + | - |
| Ryan et al. | USA | COVID-19 Community Stabilization and Sustainability Framework: An Integration of the Maslow Hierarchy of Needs and Social Determinants of Health | Disaster Medicine and Public Health Preparedness | general SDoH, income, occupation | Primary: protective measures | + | + | + | + | + | + |
| Salazar Mather et al. | USA | Love in the time of COVID-19: negligence in the Nicaraguan response | The Lancet Global Health | governance/ policy, income (LMIC), governance/ policy (healthcare system capacity) | Primordial: advocacy  Primary: protective measures | + | + | + | + | + | + |
| Santos | Portugal | Reflections about the impact of the SARS-COV-2/ COVID-19 pandemic on mental health | Brazilian Journal of Psychiatry | mental health disorder, occupation | Primary: unintended consequences | + | + | + | + | + | + |
| Sasangohar et al. | USA | Provider Burnout and Fatigue During the COVID-19 Pandemic: Lessons Learned From a High-Volume Intensive Care Unit | Anesthesia and analgesia | occupation, income | Primary: unintended consequences | + | + | + | + | + | + |
| Sattar,  McInnes &  McMurray | UK | Obesity a Risk Factor for Severe COVID-19 Infection: Multiple Potential Mechanisms | Circulation | general SDoH, race/ ethnicity | None | + | + | + | + | + | + |
| Schlögl &  A. Jones | Switzerland, USA | Maintaining Our Humanity Through the Mask: Mindful Communication During COVID-19 | Journal of the American Geriatrics Society | age (elderly), disability | Primary: communication and education | + | + | + | + | + | - |
| Schrack,  Wanigatunga, & Juraschek | USA | After the COVID-19 Pandemic: The Next Wave of Health Challenges for Older Adults | The journals of gerontology. Series A, Biological sciences and medical sciences | age (elderly), social isolation, income, food security | Primordial: research  Primary: telehealth, | + | + | + | + | + | + |
| Semple &  Cherrie | UK | Covid-19: Protecting Worker Health | Annals of Work Exposures and Health | occupation | Primordial: research  Primary: protective measures | + | + | + | + | + | + |
| Setiati &  Azwar | Indonesia | COVID-19 and Indonesia | Acta Med Indones-Indonesian Journal of Internal Medicine | governance/ policy (healthcare system capacity) | Primary: protective measures, quarantine, unintended consequences  Secondary: COVID-19 testing, isolation | + | + | + | + | + | - |
| Shah, J. et al. | USA, Jordan, Afghanistan, Iran, Japan | COVID-19: the current situation in Afghanistan | The Lancet Global Health | governance/ policy (healthcare system capacity), income (LMIC), immigrant/ refugee, education | Primordial: policy  Primary: communication and education | + | + | + | + | + | - |
| Shah, M.,  Sachdeva &  Dodiuk-Gad | Canada, Israel | COVID-19 and racial disparities | Journal of the American Academy of Dermatology | race/ ethnicity, occupation, income | Primordial: research | + | + | + | + | + | ? |
| Shalev &  Shapiro | USA | Epidemic psychiatry: The opportunities and challenges of COVID-19 | General Hospital Psychiatry | mental health disorder, housing (homeless), income | Primordial: advocacy  Primary: communication and education, protective measures, unintended consequences  Tertiary: supports | + | + | + | + | + | + |
| Sharma,  Lawrence &  Giovinazzo | UK, Italy | Transplant programs during COVID-19: Unintended consequences for health inequality | American Journal of Transplantation | race/ ethnicity, immigrant/ refugee | Primary: communication and education | + | + | + | + | + | + |
| Shiau et al. | USA | The Burden of COVID-19 in People Living with HIV: A Syndemic Perspective | AIDS and Behavior | mental health disorder, income, race/ ethnicity, food security | Primordial  Primary: unintended consequences | + | + | + | + | + | + |
| Shigemura et al. | Japan | Public responses to the novel 2019 coronavirus (2019-nCoV) in Japan: Mental health consequences and target populations | Psychiatry and Clinical Neurosciences | general SDoH, race/ ethnicity, mental health disorder, occupation | Primordial: advocacy | + | + | + | + | + | - |
| Shimizu | UK | 2019-nCoV, fake news, and racism | The Lancet | race/ ethnicity | Primary: communication and education | + | + | + | + | + | - |
| Shoptaw,  Goodman-Meza &  Landovitz | USA | Collective Call to Action for HIV/AIDS Community-Based Collaborative Science in the Era of COVID-19 | AIDS and Behavior | general SDoH, mental health (substance use), housing | Primary: unintended consequences | + | + | + | + | + | + |
| Sim | Australia | The COVID-19 pandemic: Major risks to healthcare and other workers on the front line | Occupational and Environmental Medicine | occupation | Primordial: research | + | + | + | + | + | - |
| Simpson &  Butler | Australia | Covid-19, prison crowding, and release policies | The BMJ | housing (prison) education, mental health disorder (substance use) | Primary | + | + | + | + | + | + |
| Smith, J. & Judd | Australia | COVID-19: Vulnerability and the power of privilege in a pandemic | Health Promotion Journal of Australia | general SDoH, age(elderly), race/ ethnicity, housing (prison), income (health insurance) | Primordial: policy  Primary: communication and education | + | + | + | + | + | + |
| Smith, G., Ng & Li | Hong Kong | COVID-19: Emerging compassion, courage and resilience in the face of misinformation and adversity | Journal of Clinical Nursing | race/ ethnicity, occupation | Primary: unintended consequences | + | + | + | + | - | ? |
| Smith, K., Ostinelli &  Cipriani | UK | Covid-19 and mental health: A transformational opportunity to apply an evidence-based approach to clinical practice and research | Evidence-Based Mental Health | mental health disorder, social isolation | Primordial: research | + | + | + | + | + | ? |
| Sodhi | USA | Telehealth Policies Impacting Federally Qualified Health Centers in Face of COVID-19 | Journal of Rural Health | general SDoH, rural/ urban, gender, income | Primordial: policy  Primary: telehealth | + | ? | + | + | - | - |
| Solomon, H. | USA | COVID-19 checklist: Mask, gloves, and video chatting with grandpa | Psychiatry Research | social isolation, age (elderly), mental health disorder | Primary: unintended consequences | + | + | + | + | + | + |
| Solomon, M.,  Wynia &  Gostin | USA | Scarcity in the Covid-19 Pandemic | Hastings Center Report | income (LMIC), occupation | Primary: protective measures | + | ? | + | + | - | - |
| Sood et al. | USA | Caring for Miners During the Coronavirus Disease-2019 (COVID-19) Pandemic | Journal of Rural Health | occupation, social isolation, income (health insurance), rural/ urban, disability | Primary: communication and education | + | + | + | + | + | + |
| Steinman,  Perry &  Perissinotto | USA | Meeting the Care Needs of Older Adults Isolated at Home during the COVID-19 Pandemic | JAMA Internal Medicine | age (elderly), social isolation, income, mental health disorder | Primary: protective measures, unintended consequences | + | + | + | + | + | + |
| Sun et al. | China, USA, UK | Editorial: Challenges to Opioid Use Disorders During COVID-19 | American Journal on Addictions | mental health disorder (substance use) | Primordial: research  Primary: protective measures, unintended consequences  Tertiary: supports | + | + | + | + | + | + |
| Sun, Bao & Lu | China | Addressing mental health care for bereavements during COVID-19 pandemic | Psychiatry and Clinical Neurosciences | mental health disorder, general SDoH | Tertiary: supports | + | + | + | + | + | + |
| Tandon | India | COVID-19: Impact on health of people &amp; wealth of nations | Indian Journal of Medical Research | income (LMIC) | Primordial: policy, research  Primary: communication and education | + | ? | + | + | + | + |
| Tang, Gaoshan &  Ahonsi | China, Switzerland | Sexual and reproductive health (SRH): A key issue in the emergency response to the coronavirus disease (COVID-19) outbreak | Reproductive Health | gender | Primordial: policy, research | + | + | + | + | + | + |
| Tapper &  Asrani | USA | The COVID-19 pandemic will have a long-lasting impact on the quality of cirrhosis care | Journal of Hepatology | social isolation, food security, mental health disorder (substance use), income (health insurance), governance/ policy (healthcare system capacity) | Primordial: policy  Primary: communication and education, unintended consequences  Other | + | + | + | + | + | + |
| The Editors of Alzheimer's & Dementia | USA | Alzheimer's Disease Research Enterprise in the Era of COVID-19/SARS-CoV-2 | Alzheimer's and Dementia | age (elderly), governance/ policy (healthcare system capacity) | Primary: communication and education, protective measures | - | + | + | + | - | - |
| The Lancet | Global | Redefining vulnerability in the era of COVID-19 | The Lancet Global Health | general SDoH, housing, age, mental health disorder, income | Primordial: advocacy | - | ? | + | + | - | - |
| The Lancet | Global | COVID-19: learning from experience | The Lancet | general SDoH, governance/ policy (healthcare system capacity), housing, income, occupation | Primordial: policy | - | ? | + | + | - | - |
| The Lancet | Global | The gendered dimensions of COVID-19 | The Lancet | gender | Primordial: research | - | ? | + | + | - | - |
| The Lancet | Global | India under COVID-19 lockdown | The Lancet | income (LMIC), occupation, housing, race/ ethnicity | Primordial: policy  Primary: communication and education  Secondary: COVID-19 testing | - | ? | + | + | - | - |
| The Lancet | Global | COVID-19 will not leave behind refugees and migrants | The Lancet | immigrant/ refugee, income (LMIC), governance/ policy (healthcare system capacity) | Primordial: advocacy | - | ? | + | + | - | - |
| The Lancet Infectious Diseases | Global | COVID-19: endgames | The Lancet Infectious Diseases | income (LMIC), general SDoH | Primary: protective measures  Secondary: COVID-19 testing | - | ? | - | + | - | - |
| Thomson | UK | The COVID-19 Pandemic: A Global Natural Experiment | Circulation | social isolation, gender | Primordial: research | + | + | - | - | - | ? |
| Torous et al. | USA, UK | Digital mental health and COVID-19: Using technology today to accelerate the curve on access and quality tomorrow | Journal of Medical Internet Research | general SDoH, income, race/ ethnicity, housing | Primary: unintended consequences | + | + | + | + | + | + |
| Torres &  Sacoto | Ecuador | Localising an asset-based COVID-19 response in Ecuador | The Lancet | governance/ policy (healthcare system capacity), race/ ethnicity, immigrant/ refugee | Primary: communication and education  Secondary: COVID-19 testing, contact tracing, isolation | + | + | + | + | + | - |
| Trabacca &  Russo | Italy | Covid-19 and child disabilities: whom to protect and how | European journal of physical and rehabilitation medicine | disability, age (pediatric), social isolation | Primary: unintended consequences | + | + | + | + | - | - |
| Troyer,  Kohn &  Hong | USA | Are we facing a crashing wave of neuropsychiatric sequelae of COVID-19? Neuropsychiatric symptoms and potential immunologic mechanisms | Brain, Behavior, and Immunity | mental health disorder, social isolation | Primordial: research  Primary: unintended consequences | + | + | + | + | + | + |
| Tsai &  Wilson | USA | COVID-19: a potential public health problem for homeless populations | The Lancet Public Health | housing (homeless), mental health disorder, mental health disorder (substance use) | Primary: communication and education, quarantine  Secondary: COVID-19 testing | + | + | + | + | + | + |
| Tseng et al. | Taiwan | The Impact of the COVID-19 Pandemic on Disabled and Hospice Home Care Patients | The journals of gerontology. Series A, Biological sciences and medical sciences | disability | Primary: communication and education, protective measures, unintended consequences | + | + | + | + | + | + |
| Usher et al. | Australia | Family violence and COVID-19: Increased vulnerability and reduced options for support | International Journal of Mental Health Nursing | gender, age (pediatric), income, social isolation | Primary: unintended consequences | + | + | + | + | + | + |
| Usher,  Bhullar &  Jackson | Australia | Life in the pandemic: Social isolation and mental health | Journal of Clinical Nursing | age, income, gender, mental health disorder, race/ ethnicity | Primordial: advocacy | + | + | + | + | + | - |
| Usher, Durkin &  Bhullar | Australia | The COVID-19 pandemic and mental health impacts | International Journal of Mental Health Nursing | race/ ethnicity, occupation | Primary: unintended consequences | + | + | + | + | + | + |
| Van den Broucke | Belgium | Why health promotion matters to the COVID-19 pandemic, and vice versa | Health Promotion International | age (elderly), disability, immigrant/ refugee | Primordial: policy  Primary: communication and engagement  Other | + | + | + | + | + | + |
| van der Werf & Peltekian | France | Facing challenges with the novel coronavirus SARS-CoV-2 outbreak | Virologie | income (LMIC), governance/ policy (healthcare system capacity) | Primordial: research | + | + | + | + | + | - |
| van Gelder et al. | Netherlands, USA, Italy, Germany | COVID-19: Reducing the risk of infection might increase the risk of intimate partner violence | EClinicalMedicine | gender, social isolation, age (pediatric) | Primary: communication and education, unintended consequences | + | + | + | + | + | + |
| Van Lancker & Parolin | Belgium, USA | COVID-19, school closures, and child poverty: a social crisis in the making | The Lancet Public Health | age (pediatric), income, food security | Primary: unintended consequences | + | + | + | + | + | + |
| Vanderpuye,  Elhassan &  Simonds | Ghana, Sudan, South Africa | Preparedness for COVID-19 in the oncology community in Africa | The Lancet Oncology | governance/ policy (healthcare system capacity), income (LMIC), income, education | None | + | + | + | + | + | + |
| Venkatesh &  Edirappul | UK | Social distancing in covid-19: what are the mental health implications? | The BMJ | mental health disorder | Primary: unintended consequences | + | + | + | + | + | - |
| Vessey &  Betz | USA | Everything old is new again: COVID-19 and public health | Journal of Pediatric Nursing | age (pediatric), income, education | Primordial: advocacy | + | + | + | + | + | + |
| Vieira et al. | Switzerland, Columbia | COVID-19: The forgotten priorities of the pandemic | Maturitas | general SDoH, income, immigrant/ refugee, race/ ethnicity, housing | Primary: communication and education, unintended consequences | + | + | + | + | + | + |
| Volkow | USA | Collision of the COVID-19 and Addiction Epidemics | Annals of Internal Medicine | mental health disorder (substance use), housing | Primordial: advocacy | + | + | + | + | + | - |
| Wagner | USA | Addressing the experience of children and adolescents during the CoviD-19 pandemic | Journal of Clinical Psychiatry | age (pediatric), income, food security | Primary: communication and education | + | ? | + | - | - | - |
| Walensky &  Del Rio | USA | From Mitigation to Containment of the COVID-19 Pandemic: Putting the SARS-CoV-2 Genie Back in the Bottle | JAMA | income, housing, occupation | Primordial  Secondary: COVID-19 testing | + | + | - | + | - | - |
| Walter &  McGregor | USA | Sex- And gender-specific observations and implications for COVID-19 | Western Journal of Emergency Medicine | gender | Primordial: research | + | + | + | + | + | + |
| Walter-McCabe | USA | Coronavirus Pandemic Calls for an Immediate Social Work Response | Social Work in Public Health | general (SDoH), income, disability, housing (prison), occupation | Primordial: advocacy, policy | + | + | + | + | + | - |
| Wang, H. et al. | China, Spain, Canada, Australia | Dementia care during COVID-19 | The Lancet | age (elderly), social isolation | Primary: unintended consequences | + | + | + | + | + | - |
| Wang, Z. &  Tang | China | Combating COVID-19: health equity matters | Nature Medicine | income, age (elderly) | Primordial: policy, research | + | + | + | + | + | - |
| Weng et al. | Taiwan | New preventative measures against coronavirus disease 2019 for home care aides in Taiwan | The journals of gerontology. Series A, Biological sciences and medical sciences | housing (LTC) | Primary: quarantine, protective measures, unintended consequences | + | + | + | + | + | - |
| Wenham,  Smith &  Morgan | UK, Canada, USA | Covid-19 is an opportunity for gender equality within the workplace and at home | The BMJ | gender, occupation | Primordial | + | + | + | + | - | + |
| Wenham,  Smith &  Morgan | Canada | COVID-19: the gendered impacts of the outbreak | The Lancet | gender | Primordial: advocacy, research | + | + | + | + | + | + |
| Whaibeh,  Mahmoud &  Naal | Lebanon, USA | Telemental Health in the Context of a Pandemic: the COVID-19 Experience | Current Treatment Options in Psychiatry | mental health disorder | Primordial: policy  Primary: communication and education, telehealth, unintended consequences | + | ? | + | + | - | + |
| White & Lo | USA | A Framework for Rationing Ventilators and Critical Care Beds during the COVID-19 Pandemic | JAMA | governance/ policy (healthcare system capacity) | Primordial: policy | + | + | + | + | + | + |
| Whitworth | UK | COVID-19: A fast evolving pandemic | Transactions of the Royal Society of Tropical Medicine and Hygiene | income (LMIC) | Primordial: policy, research  Primary: communication and education | + | + | + | + | - | - |
| Williamson,  Murphy &  Greenberg | UK | COVID-19 and experiences of moral injury in front-line key workers | Occupational Medicine | occupation | Primary: unintended consequences | + | + | + | + | + | - |
| Wilson,  Ramage &  Fagan | USA | A Primary Care Response to COVID-19 for Patients with an Opioid Use Disorder | Journal of Rural Health | mental health disorder (substance use), rural/ urban, housing (homeless) | Primordial: advocacy | + | + | + | + | + | + |
| Wise | USA | Implications of COVID-19 for Primary Care | Primary Care Reports | race/ ethnicity | Primary: communication and education, telehealth | + | ? | + | - | + | - |
| Wong et al. | USA | Mitigating the Impacts of the COVID-19 Pandemic Response on At-Risk Children | Pediatrics | age (pediatric), income | Primary: unintended consequences | + | + | + | + | + | + |
| Wood, Davies &  Khan | Australia, UK | COVID-19 precautions: easier said than done when patients are homeless | Medical Journal of Australia | housing (homeless) | Primordial: advocacy | + | + | + | + | + | + |
| Wosik et al. | USA | Telehealth Transformation: COVID-19 and the rise of Virtual Care | Journal of the American Medical Informatics Association | occupation, social isolation | Primordial: policy | + | + | + | + | + | ? |
| Wurcel et al. | USA | Spotlight on Jails: COVID-19 Mitigation Policies Needed Now | Clinical Infectious Diseases | housing (prison) | Primordial: policy  Primary: communication and education, protective measures  Secondary: COVID-19 testing, isolating | + | + | + | + | + | - |
| Xiang et al. | China, Australia | Timely mental health care for the 2019 novel coronavirus outbreak is urgently needed | The Lancet Psychiatry | occupation, mental health disorder | Primary: communication and education, unintended consequences | + | + | + | + | + | - |
| Yamey et al. | USA, Germany, UK | Ensuring global access to COVID-19 vaccines | The Lancet | income (LMIC) | Primary: communication and education | + | + | + | + | + | ? |
| Yancy | USA | COVID-19 and African Americans | JAMA | race/ ethnicity, housing, food security, income | Primordial: advocacy | + | + | + | + | + | + |
| Yang &  Thompson | UK | Fighting covid-19 outbreaks in prisons | The BMJ | housing (prison) | Primordial: policy  Primary: communication and education | + | ? | + | + | + | - |
| Yao, Chen & Xu | USA, China, UK | Patients with mental health disorders in the COVID-19 epidemic | The Lancet Psychiatry | mental health disorder | Primordial: advocacy | + | + | + | + | + | - |
| Yen et al. | Taiwan | Recommendations for protecting against and mitigating the COVID-19 pandemic in long-term care facilities | Journal of Microbiology, Immunology and Infection | age, age (elderly) | Primordial: policy  Primary: communication and education, quarantine, protective measures  Secondary: isolation | + | + | + | + | + | + |
| Yip & Chau | Hong Kong | Physical Distancing and Emotional Closeness Amidst COVID-19 | Crisis | age (elderly) | Primary: unintended consequences | + | + | + | + | + | - |
| Young &  Fick | USA | Public Health and Ethics Intersect at New Levels With Gerontological Nursing in COVID-19 Pandemic | Journal of Gerontological Nursing | age (elderly), social isolation, housing (LTC) | Primary: unintended consequences  Tertiary: supports | + | + | + | + | + | - |
| Zandifar &  Badrfam | Iran | Iranian mental health during the COVID-19 epidemic | Asian Journal of Psychiatry | social isolation | Primary: unintended consequences | + | + | + | ? | + | - |
| Zavaleta | Peru | COVID-19: protect Indigenous peoples | Nature | race/ ethnicity | Primordial: advocacy, research | + | ? | + | - | - | - |
| Zhai & Du | USA | Addressing collegiate mental health amid COVID-19 pandemic | Psychiatry Research | mental health disorder, social isolation, education | Primary: unintended consequences | + | ? | + | + | + | ? |
| Zhang et al | Hong Kong, USA, Switzerland, Netherlands, China | The Novel Coronavirus Outbreak: What We Know and What We Don't | Cell | income (LMIC) | Other | + | + | + | + | - | - |
| Zheng | China | Mental health and a novel coronavirus (2019-nCoV) in China | Journal of Affective Disorders | occupation | None | + | + | + | + | + | - |
| Zhu et al. | China | The Risk and Prevention of Novel Coronavirus Pneumonia Infections Among Inpatients in Psychiatric Hospitals | Neuroscience Bulletin | mental health disorder, housing, occupation | Primary: communication and education  Secondary: isolation | + | + | + | + | + | - |
| Zimmerman et al. | USA | The Need to Include Assisted Living in Responding to the COVID-19 Pandemic | Journal of the American Medical Directors Association | housing (LTC) | Primordial: policy  Primary: communication and education, protective measures, unintended consequences  Secondary: isolation | + | + | + | + | + | + |

**Quality Appraisal (QA) Legend**

QA1: Is the source of the opinion clearly identified?

QA2: Does the source of opinion have standing in the field of expertise?

QA3: Are the interests of the relevant population the central focus of the opinion?

QA4: Is the stated position the result of an analytical process, and is there logic in the opinion expressed?

QA5: Is there reference to the extant literature?

QA6: Is any incongruence with the literature/sources logically defended?

+ = yes

- = no

? = unclear

/ = not applicable
